# Supplementary material for: Patient data-sharing for immigration enforcement: a qualitative study of healthcare providers in England
Source: BMJ Open. 2020 Feb 12;10(2):e033202. doi: 10.1136/bmjopen-2019-033202 (PMC7044876; doi:10.1136/bmjopen-2019-033202)
Supplement: Supplementary data [file bmjopen-2019-033202supp001.pdf]

Supplementary Appendix 1: Non-clinical data shared in accordance to the MoU, including if individual is deceased. Source: NHS Digital.

Non-clinical data shared

- Name (and date last known to be effective)
- Gender
- Date of birth (and date last known to be effective)
- Address
- Primary care support area code
- Primary care support area name
- Date of GP/primary care registration
- Primary care support England contact details (email and telephone number)

Data shared (if deceased)

- Name (and date last known to be effective)
- Date of birth
- Death registration district
- Death registration year/quarter

REFERENCE: NHS Digital. Written evidence from NHS Digital (MOU0004): House of Commons, 2018.
