# Supplementary material for: Patient data-sharing for immigration enforcement: a qualitative study of healthcare providers in England
Source: BMJ Open. 2020 Feb 12;10(2):e033202. doi: 10.1136/bmjopen-2019-033202 (PMC7044876; doi:10.1136/bmjopen-2019-033202)
Supplement: Supplementary data [file bmjopen-2019-033202supp002.pdf]

Supplementary Appendix 2: Interview discussion topic guide

<u>Introductory Questions</u>

1. Can you please tell me more about your organisation and your role?

2. How many years have you been working in this role?

3. How many years have you been practicing medicine?

<u>Theme 1: Awareness, understanding, perceptions of patient data sharing, as described in the Home Office, Department of Health, NHS Digital MoU</u>

1. Does your practice /organisation collect demographic data about people who come to access services? If so, please could you tell me the type of data that is collected and recorded? (e.g. name, DOB, country of birth, NHS number, current address (is this mandatory?), telephone number)

2. Outside of the NHS system, who may have access to patient demographic data?

3. Who do you think should have access to patient demographic data, outside of the NHS? Prompts: Why? Why Not?

4. What can you tell me about NHS Digital sharing data with the Home Office?

ONLY *if they* ***do not know anything*** *about it, read out: "Since 2013, NHS Digital had been informally sharing patient administrative data, such as name, date of birth and address with the Home Office on an ad hoc basis to assist the Home Office with locating and prosecuting migrants in relation to immigration issues. That process was formalised in a Memorandum of Understanding in January 2017. This agreement was amended in May 2018. "*

a. How did you hear about this data sharing arrangement?

b. When do you first remember hearing about it?

c. What do you think about this policy? (of NHS Digital sharing demographic data with the Home Office in order for the Home Office to investigate immigration offences; previously any offenders but now only individuals convicted of, or under investigation for, serious crime?)

5. Has your awareness about this data sharing influenced how your practice / organisation records patient administrative data? If so, how?

6. Has knowledge of the data sharing changed the advice you give to patients? If so, how?

7. How was the data sharing viewed by your colleagues? Organisation? and peers?

8. Can you tell me about any interactions that you have had with patients about the data sharing?

9. Based on your experience, did knowledge that administrative data was being shared between NHS Digital and the Home Office have implications for how patients felt about accessing your services? How so?

Theme 2: Experience with migrant patients

10. In your work as XXX (e.g. GP), do you see patients who have been born in a country other than the UK, i.e. migrants?

11. Can you tell me more about this patient population? Prompts: Country of origin, English language ability

12. What have your experiences been like with migrant patients? (i.e. compared to UK born patients)?

13. From your experience, what can you tell me about their uptake of primary health services? Compared to British patients?

14. In your experience, is there a distinct different with migrant patients' health needs?

15. Thinking about your own experience, are you aware of any barriers that migrants might face in accessing services? Can you expand on what these might be and why?

16. From your experience, have migrant patients expressed any concerns about their data being shared with the Home Office, or concerns that accessing health services may lead to immigration authorities contacting them?

17. Thinking about this issue, has the data sharing affected your relationship with migrant patients?

Theme 3: Patient confidentiality

18. As a clinician, how you do view this data sharing in relation to patient confidentiality? Prompts: Why? How?

19. How do you think the NHS Digital data sharing may be perceived by the public? Specifically, for example public trust in NHS' management and guardianship of patient data?

Theme 4: Patient safety

IF participant cites fear of immigration or data sharing as a barrier for migrants' uptake of health services:

20. In your opinion, what are the implications of the data sharing for patient safety? For migrant patients? For vulnerable populations such as pregnant women, children, people with infectious diseases?

21. Lastly, what do you think of the data sharing in the broader terms of the general public health?

Thank you for your time. Do you have any questions for me?

****END RECORDING****
