# Supplementary material for: Patient data-sharing for immigration enforcement: a qualitative study of healthcare providers in England
Source: BMJ Open. 2020 Feb 12;10(2):e033202. doi: 10.1136/bmjopen-2019-033202 (PMC7044876; doi:10.1136/bmjopen-2019-033202)
Supplement: Supplementary data [file bmjopen-2019-033202supp003.pdf]

Supplementary Appendix 3: The 7 NHS Principles of the NHS Constitution for England. Source: Department of Health and Social Care.

NHS Principle	Summarised term
1. Provide comprehensive service, available to all	Universal access (equality)
2. Access based on clinical need, not individual's ability to pay	Needs-based
3. Aspires to the highest standards of excellence and professionalism	Quality of care
4. Patient at the heart of everything NHS does	Patient-centricity
5. Working across organisational boundaries	Collaboration
6. Provides best value for taxpayer's money	(Economic) Efficiency
7. Accountable to public, communities and patients it serves	Accountability

REFERENCE: Department of Health and Social Care. *The NHS Constitution for England*. [Updated October 2015] Available from: https://www.gov.uk/government/publications/the-nhsconstitution-for-england/the-nhs-constitution-for-england [Accessed: 17th May 2018].
