# Supplementary material for: Patient data-sharing for immigration enforcement: a qualitative study of healthcare providers in England
Source: BMJ Open. 2020 Feb 12;10(2):e033202. doi: 10.1136/bmjopen-2019-033202 (PMC7044876; doi:10.1136/bmjopen-2019-033202)
Supplement: Author's manuscript [file bmjopen-2019-033202.draft_revisions.pdf]

BMJ Open

BMJ Open is committed to open peer review. As part of this commitment we make the peer review history of every article we publish publicly available.

When an article is published we post the peer reviewers' comments and the authors' responses online. We also post the versions of the paper that were used during peer review. These are the versions that the peer review comments apply to.

The versions of the paper that follow are the versions that were submitted during the peer review process. They are not the versions of record or the final published versions. They should not be cited or distributed as the published version of this manuscript.

BMJ Open is an open access journal and the full, final, typeset and author-corrected version of record of the manuscript is available on our site with no access controls, subscription charges or pay-per-view fees (http://bmjopen.bmj.com).

If you have any questions on BMJ Open's open peer review process please email info.bmjopen@bmj.com

BMJ Open

Patient data sharing for immigration enforcement: a qualitative study of healthcare providers in England

Journal:	*BMJ Open*
Manuscript ID	bmjopen-2019-033202
Article Type:	Research
Date Submitted by the Author:	25-Jul-2019

Complete List of Authors:	Papageorgiou, Vasiliki; Imperial College London School of Public Health, Patient Experience Research Centre<br>Wharton-Smith, Alexandra; London School of Hygiene and Tropical Medicine Faculty of Public Health and Policy, Department of Global Health and Development<br>Campos-Matos, Ines; Public Health England, Migration Health, Health Improvement Directorate<br>Ward, Helen; Imperial College London School of Public Health, Patient Experience Research Centre

Keywords:	Health policy < HEALTH SERVICES ADMINISTRATION & MANAGEMENT, MEDICAL ETHICS, QUALITATIVE RESEARCH, Migrant Health, PUBLIC HEALTH

SCHOLARONE™
Manuscripts

I, the Submitting Author has the right to grant and does grant on behalf of all authors of the Work (as defined in the below author licence), an exclusive licence and/or a non-exclusive licence for contributions from authors who are: i) UK Crown employees; ii) where BMJ has agreed a CC-BY licence shall apply, and/or iii) in accordance with the terms applicable for US Federal Government officers or employees acting as part of their official duties; on a worldwide, perpetual, irrevocable, royalty-free basis to BMJ Publishing Group Ltd ("BMJ") its licensees and where the relevant Journal is co-owned by BMJ to the co-owners of the Journal, to publish the Work in this journal and any other BMJ products and to exploit all rights, as set out in our licence.

The Submitting Author accepts and understands that any supply made under these terms is made by BMJ to the Submitting Author unless you are acting as an employee on behalf of your employer or a postgraduate student of an affiliated institution which is paying any applicable article publishing charge ("APC") for Open Access articles. Where the Submitting Author wishes to make the Work available on an Open Access basis (and intends to pay the relevant APC), the terms of reuse of such Open Access shall be governed by a Creative Commons licence – details of these licences and which Creative Commons licence will apply to this Work are set out in our licence referred to above.

Other than as permitted in any relevant BMJ Author's Self Archiving Policies, I confirm this Work has not been accepted for publication elsewhere, is not being considered for publication elsewhere and does not duplicate material already published. I confirm all authors consent to publication of this Work and authorise the granting of this licence.

For peer review only

**Title**	**Patient data sharing for immigration enforcement: a qualitative study of healthcare providers in England**
**Author's names**	Vasiliki Papageorgiou (0000-0002-2387-6780), Alexandra Wharton-Smith (0000-0001-5335-2059), Ines Campos-Matos (0000-0002-6480-4310), Helen Ward (0000-0001-8238-5036)
**Address for each author, authors' names and positions**	• Research Assistant, Patient Experience Research Centre, School of Public Health, Imperial College London, UK<br>• PhD Candidate, Department of Global Health and Development, Faculty of Public Health and Policy, London School of Hygiene and Tropical Medicine, UK<br>• Consultant Epidemiologist, Migration Health, Health Improvement Directorate, Public Health England, UK<br>• Professor of Public Health, Patient Experience Research Centre, School of Public Health, Imperial College London, UK
**Corresponding author**	Correspondence to:<br>Vasiliki Papageorgiou, vasiliki.papageorgiou17@imperial.ac.uk
**Word count**	3754 words

For peer review only

ABSTRACT

Aim: To explore healthcare providers' perceptions and experiences of the implications of a patient data sharing agreement between NHS Digital and the Home Office on access to NHS services and quality of care received by migrant patients in England.

Design: A qualitative study using semi-structured interviews, thematic analysis and constant-comparison approach.

Participants: 12 healthcare providers working in community, hospital, or voluntary sector-based settings who had experience of migrants accessing NHS England services. Interviews were carried out in 2018.

Setting: England.

Results: Awareness and understanding of the patient data sharing agreement varied among participants, who associated this with a perceived lack of transparency by government. Participants provided insight into how they thought the data sharing agreement was negatively influencing migrants' health-seeking behaviour, their relationship with clinicians and the safety and quality of their care. They referred to the policy as a challenge to their core ethical principles, explicitly patient confidentiality and trust, which varied depending on their clinical specialty.

Conclusions: A perceived lack of transparency during the policy development process can result in suspicion or mistrust towards government amongst the health workforce, patients and public, which is underpinned by a notion of power or control. The patient data sharing agreement was considered a threat to some of the core principles of the NHS and its implementation as adversely affecting healthcare access and patient safety. Future policy development should involve a range of stakeholders including civil society, healthcare professionals and ethicists, and include more meaningful assessments of the impact on healthcare and public health.

Keywords: Health Policy; Medical Ethics; Migrant Health; Public Health; Qualitative Research.

For peer review only

SUMMARY

Strengths and limitations of this study

- To our knowledge, this is the first primary research study to explore the experiences and perspectives of healthcare providers in relation to the patient data sharing agreement between NHS Digital and the Home Office.
- The interviews generated large amounts of rich, contextual data with data saturation met for *a priori* themes.
- The findings were interpreted using a social constructionist approach, acknowledging the researcher's own biases through a reflexive process and discussed with others to provide additional rigour.
- The main study limitation was sample size due to the limited availability of healthcare providers and difficulties with recruitment.
- Participants were also aware of the study purpose prior to taking part which may have influenced recruitment and the findings.

For peer review only

INTRODUCTION

In 2017, a Memorandum of Understanding (referred to here as the data sharing agreement) was agreed between the UK Home Office and NHS Digital, with the Department of Health and Social Care acting as a signatory.[1] This formalised an existing arrangement, in place since 2013, whereby non-clinical patient data **(Supplementary 1)** were being shared to trace suspected immigration offenders, without patient consent nor sometimes the knowledge of clinicians; in some cases, primary care practices were asked directly by the Home Office to confirm patients' addresses.[1 2] NHS Digital ruled the process as justified as the information requested excluded clinical data and "lies at the least intrusive end of the spectrum of confidential information".[3] However, concerns were raised about the potential impact of the data sharing agreement on access to NHS services by diverse and often vulnerable migrant populations, and to the wider public health, including sexual and reproductive health.[4-7] Following these objections, an amendment was made to restrict requests to individuals being considered for deportation and convicted of 'serious' criminal offences, or presenting a risk to public security, to ensure compliance with the newly introduced General Data Protection Regulation (GDPR).[8-10] The new agreement has yet to be published however, a revision plan has been issued and the original agreement has now been withdrawn.[1 11] Recently, proposals for a similar agreement to determine health-related welfare entitlements between the NHS and Department for Work and Pensions, has also been reported.[12 13]

Migration itself has been described as a core determinant of health and wellbeing and various factors have been shown to influence the health-seeking behaviour and access to healthcare services of documented and undocumented migrants, including cultural ideas and expectations, socio-economic conditions, geographical and institutional factors.[5 14 15] However, important gaps exist in the literature on migrant health, health-seeking (or avoidance) behaviour and access generally; national and international comparisons are challenging due to differing definitions, contexts and limited epidemiological data.[16 17] For instance, research conducted in Spain has found both a higher and lower utilisation of GP services by different groups of migrants, compared to the native population, due to inequities when seeking initial contact.[18-20] Studies globally, including the United States, have demonstrated that perceived fear of deportation may act as a barrier to migrants accessing health services and has been associated with poor health outcomes including mental health.[21-25]

In England, NHS charging regulations are used to determine eligibility for free NHS services with these guidelines being reported as a barrier for migrants accessing healthcare.[5 26 27] Even where services are free, for example primary care, access may be limited by registration

For peer review only - http://bmjopen.bmj.com/site/about/guidelines.xhtml

processes and documentation required which can vary by the GP practice.[28] Additionally, undocumented migrants are often unaware of their entitlements and report mixed experiences during registration, including language barriers.[29] Consequently, there are concerns that unregistered individuals are shifting costs to secondary care due to late presentation with greater clinical severity.[30 31] Discontinuity in care, felt hostility or perceived stigma can further increase difficulties whilst navigating the health system; the healthcare system itself may act as a social determinant.[32 33] To date, most UK studies focus on specific subgroups, populations and areas within the country; for instance, Burmese migrants accessing GP services in London.[34]

Concerns have been reported about the collection of individuals' migration status in healthcare systems globally, particularly regarding the potential misuse of health data and critics emphasise that patient confidentiality and trust are incompatible with the data sharing agreement.[9 15 23 35] These concerns reflect broader political contexts; a "hostile environment" has been explicitly promoted by the UK government in relation to migration, evident in the application of immigration policy to other areas including social welfare since 2010.[36 37] This sits uncomfortably with the increased onus on public bodies to regulate health and care data, including the network of UK Caldicott Guardians responsible for managing data access requests and protecting an individual's confidentiality within NHS organisations and local authorities providing social services.[38 39] Additionally, there is limited research on the potential conflicts between these different parts of government, for example on the impact of sharing healthcare data for the specific purpose of immigration enforcement. Therefore, it is important to understand the perceptions of the agreement by healthcare workers to explore whether the policy has influenced clinical care provided and healthcare received by migrant patients and possibly decision-making processes in clinical care and healthcare data management, particularly as a new agreement is currently being drafted. This study aims to contribute to the growing evidence of predominantly grey literature[40], editorials[36] and patient case studies[35 41], by providing robust data on the experiences of the data sharing agreement among healthcare providers in England.

For peer review only - http://bmjopen.bmj.com/site/about/guidelines.xhtml

METHODS

Study design

We carried out a qualitative study using interviews with healthcare providers. The study draws on the NHS principles of the NHS Constitution for England as a framework, as previously performed by Rafighi *et al*[42], to inform their perceptions and experience of migrants' access to health services and migrants' health-seeking behaviour. The study formed the thesis for a postgraduate degree, which participants were made aware of. Ethical approval was obtained from Imperial College London (18IC4526) and Public Health England (R&D H088).

Sampling

Participants were healthcare professionals or voluntary staff in England with experience supporting migrants accessing healthcare services. Selection initially focused on primary healthcare providers and voluntary sector workers, but we expanded to include healthcare professionals in secondary care following slow initial recruitment.

Participants were recruited through existing personal contacts and professional networks (VP, HW, ICM, AWS), social media and professional bodies.

Data collection

A female, postgraduate researcher (VP) conducted semi-structured, in-depth interviews in July 2018, either in person (n=7) at the participant's workplace (non-NHS Trust site) or the university campus, or by telephone/online (n=5). Recruitment continued until theoretical saturation was met.[43]

An initial topic guide was used to encourage participants to describe their experiences and views of migrants accessing healthcare, generally and in relation to the data sharing agreement. The interview focussed on the 'how', rather than the 'what', in line with the constructionist concept of this interaction.[44] Some themes were determined *a priori*, with emergent themes explored through subsequent interviews and the relationships between concepts investigated. Four initial themes were explored in the topic guide **(Supplementary 2)** and pilot tested with HW and AWS. If participants were unaware or indicated little understanding of the patient data sharing agreement, a short description was given. Probes were used to encourage participants to elaborate their answers and descriptive field notes were recorded by VP.

All interviews were audio recorded and transcribed verbatim by VP, after participants received a participant information sheet (PIS) and provided written and oral consent.

For peer review only - http://bmjopen.bmj.com/site/about/guidelines.xhtml

Data analysis

Memos were written on transcripts by VP describing initial reflections, with the findings analysed using an inductive-deductive approach through constant comparison, which is a feature of grounded theory.[45 46] NVivo 12 was used to organise the data, with a primary thematic framework provided by the topic guide. Coding was completed by VP through initial line-by-line content analysis with subsequent focussed coding and constant-comparison between codes to generate emergent themes and sub-themes.[45] HW and AWS provided consensus on coding.

A thematic analysis, featuring summarised NHS principles **(Supplementary 3)**, was used to organise data, where appropriate. The framework was not applied to directly categorise data as this would restrict interpretation and overlook the philosophical and empirical concepts being determined.[47]

The findings were shared with Public Health England (PHE) to contribute to their review[4] commissioned by the Department of Health and Social Care, in addition to all participants as a lay summary by email. The consolidated criteria for reporting qualitative research (COREQ) checklist has been used to report study methods and findings **(Supplementary 4)**.[48]

Patient and public involvement

There were no funds or time allocated for public involvement due to the timing of this postgraduate research project.

For peer review only

RESULTS

Study population/characteristics

Twelve participants were interviewed, with most based in London **(Table 1)**. Interviews ranged from 20 to 60 minutes. Participants worked in a range of specialties: general practice (n=4), infectious diseases and/or sexual health (n=4), psychiatry (n=1), physiotherapy (n=1), paediatrics (n=1) and community engagement (n=1). Interviewees included an equal split of men and women and a range of ages and levels of experience.

Table 1: Summary of study participants' demographics

Participant ID	Age (years)	Gender	Clinical setting*	Country of birth	Location of workplace
**B01**	25-34	F	Community	UK	London
**B02**	45-54	F	Community	UK	Other
**B03**	35-44	M	Hospital	Non-UK	London
**B04**	25-34	M	Hospital	UK	London
**B05**	45-54	M	Hospital	UK	London
**B06**	25-34	M	Hospital	Non-UK	Other
**B07**	35-44	M	Community	UK	London
**B08**	55-64	M	Community	UK	London
**B09**	45-54	F	Community	Non-UK	London
**B10**	35-44	F	Community	Non-UK	London
**B11**†	65+	F	N/A – voluntary sector	-	-
**B12**	25-34	F	Hospital	UK	London

Interviewees were asked to explain what they knew about the data sharing agreement and their understanding varied (4 provided a full description, 5 little description and 3 provided no description). Overall, most participants were confused about the specifics of the policy. Participants expressing some understanding often alluded to having learnt about the agreement either through the media or from colleagues making any subsequent answers an immediate reflection, rather than a considered opinion.

The analysis **(Figure 1)** identified three major themes: (1) patient experience, (2) ethical principles of public health and (3) power and relationships, which encompassed the themes explored in the topic guide. The themes interconnected with perceived consequences of the agreement and followed the natural sequence of the patient pathway from accessing to receiving healthcare in the NHS. This included reflections on any changes to clinical practice delivered and the patient-healthcare professional relationship.

Abbreviations: F, Female; M, Male; N/A, Not Applicable; UK, United Kingdom.
* Community (self-referral, primary care and sexual health services) or hospital settings.
† Incomplete data received.

For peer review only - http://bmjopen.bmj.com/site/about/guidelines.xhtml

Theme 1: Migrant Patient Experience of Accessing Healthcare

Healthcare providers described their interaction with migrant patients along the care pathway, although confusion about the asylum process, healthcare entitlements and definition of migrant were often described, either as a reflection of their own, or colleagues', practice.

Migrant health-seeking behaviour

Participants described a range of influences on migrants' health-seeking behaviour, with some directly related to enforcement and data sharing while others concerned broader aspects. More than half described discriminatory practices in relation to proof of eligibility:

> *"…informally we sort of found out the people with 'foreign-sounding' names were being targeted for these letters [from NHS Spine portal to confirm their home address if they had not used an NHS service in 6-months]."* **[B02]**

This linked to suggestions that migrants would experience fear and anxiety around registration and accessing health services. Specific references were made to immigration control and patients' fears of arrest or deportation of either themselves, or others in their immediate network, by the Home Office:

> *"…I think there is a group of people that are afraid and think that by coming into contact with services, they could […] put themselves on the radar."* **[B05]**

Participants talked about how these resultant fears of accessing health services could be particularly harmful for vulnerable people, for example those affected by mental illness. This reflects the backgrounds and interests of some interviewees; one described a suicidal UK citizen detained under the Mental Health Act whose husband was at risk of deportation and had received a letter from the Home Office:

> *"…we continuously sort of had to reaffirm the limits of the information that we share and how much of it is strictly confidential...."* **[B06]**

Other perceived influences on health-seeking behaviour included: attitudes to health, cultural ideas and expectations of healthcare services. Participants described how these may result in either delayed presentation or the overuse of healthcare which could influence public health funding and access to healthcare. For instance, participants working in sexual health and

For peer review only - http://bmjopen.bmj.com/site/about/guidelines.xhtml

infectious disease services described how delayed or deferred clinical presentation could shift expenses from primary to secondary care:

> *"…if you delay somebody coming forward for HIV care, they're more likely to end up needing an in-patient admission which is more expensive…"* **[B07]**

Figure 2 shows the patient pathway and potential barriers migrants may face when accessing healthcare, which are also influenced by wider determinants. These were identified from the literature and further explored, in relation to the data sharing agreement, during interviews.

Theme 2: Ethical Principles of Public Health

Transparency

Most participants described migrant patients and colleagues as being unaware and having a limited understanding of the policy. They linked this to a "sneaky" agenda, or evasion of transparency by the Home Office to access data, without patients' nor clinicians' knowledge:

> *"…GPs have no idea that this precious data they hold is being accessed, sort of on the sly, without them even realising…"* **[B01]**

Concerns were also raised about how patients first hear about the agreement which could generate fear. A non-clinical specialist working for a community-based organisation supporting marginalised populations offered insight into knowledge exchange by rumours, which may influence clinical presentation:

> *"…they will go and share that will all their friends, family […] and they will panic."* **[B11]**

Patient confidentiality and consent

Participants felt that the data sharing agreement directly contradicted the ethical principles of confidentiality and consent that underpinned the professional guidelines they abided by, resulting in an "erosion in trust in healthcare" **[B03].** One question asked for participants' opinion of the policy in relation to patient confidentiality; four described practices they had taken or were prepared to take to protect confidentiality, which was in line with advice given by Doctors of the World[49] such as changes in recording patient data by keeping separate notes, or writing in shorthand:

For peer review only - http://bmjopen.bmj.com/site/about/guidelines.xhtml

> *"…then you'd be thinking […] 'I better not put that [telephone] number in the actual [notes], I'm gonna keep that'…Maybe it's over the top but it does create this kind of space for suspicion."* **[B04]**

<u>*Non-maleficence (patient and public safety)*</u>

Some participants suggested how migrant patients may bypass the agreement by giving false information or pretending to be someone else which could impact both individual patient safety and that of the wider public. **B04** describes a case shared by a colleague where this had occurred, and its implications:

> *"…there was a pregnant woman who came to deliver her baby and she was so worried about it she […] used her sister's details... The time that they knew that, was the time they came to give her a blood transfusion, and they realised that her blood group was different…"* **[B04]**

The concept of safety was further explained from the perspective of health protection and public security. Participants reflected on the public safety rationale of the policy, following the recent amendment, offering differing views, with some being less definitive:

> *"From an infectious disease, public health prevention point of view, 'are the people that are likely to have like transmissible illnesses more likely to be the type of people that have committed serious offences?' No…"* **[B03]**

One hospital-based participant spoke of 'security' in terms of the threat of terrorism, from the perspective of having witnessed a terrorist attack, and appeared unsure, *"…I think as far as security goes… I don't know is the answer."* **[B12]**

Participants were able to appreciate "*exceptional circumstances*" **[B02]** where confidentiality may be breached, including criminal investigations or where patients may be at risk of harm to themselves or others, which some described as a "rarity" in their own experience. However, most participants disagreed with the use of the agreement for immigration purposes, often referring to the 'philosophy' or principles of the NHS or medical profession.

Theme 3: Power and relationships

<u>*Politics and policy*</u>

Participants described the political climate, without probing, referring variously to the "hostile environment", Brexit, healthcare charges or other policies they viewed as deterring migrants from accessing healthcare. One community clinician described migration as a political issue associated with "scaremongering" by the media, and some participants suggested the need

For peer review only - http://bmjopen.bmj.com/site/about/guidelines.xhtml

for governmental accountability and transparency. They proposed conducting an informed public debate on the agreement, indicating a lack of awareness of the 'open' status of the Health and Social Care Committee inquiry.[50]

Most interviewees mentioned a loss of faith in governmental decision-making processes, specifically the Home Office, following recent media attention on data sharing and immigration scandals, predominantly Windrush. The 2018 Windrush scandal refers to the generation of Caribbean-born British citizens who entered the UK over four decades ago, some of whom were wrongly deported and denied re-entry.[15 51] Some participants also raised concerns of the potential misuse of the agreement by other government agencies, for instance during benefit fraud investigations.

Relationships between patient/healthcare professionals and colleagues

Participants described trust, empathy and rapport as essential elements to the patient-healthcare professional relationship, which they perceived to be challenged by the agreement. Their narrative of the importance of this relationship were described with patients generally:

> *"…if you've got, going to have a working relationship with somebody about the most intimate details of their lives […] you have to have that trust."* **[B02]**

Participants described the positions of other colleagues on the data sharing agreement. One expressed frustration at their local Caldicott Guardian, the person with responsibility for protecting patient confidentiality and compliance with data protection regulations. When approached for guidance on the agreement within their Trust, the Caldicott Guardian had sent:

> *"…a very bland reply saying well, 'if this is what the government want to do, and they've agreed, then we'll just do it'."* **[B02]**

Conversely, **B08**, themselves a Caldicott Guardian, gave an opinion on the legality of the policy, and bemoaned lack of awareness amongst colleagues:

> *"… I believe that* **[thumps table]** *what happened here, did not follow the processes which I certainly thought was there and then, as Caldicott Guardian, thought I was part of."* **[B08]**

Participants also referred to tension between colleagues because of charging policies for overseas visitors. Two participants based in a hospital setting highlighted their negative experience of how overseas visitor officers sought to recover charges from patients. The first

For peer review only - http://bmjopen.bmj.com/site/about/guidelines.xhtml

clinician described a situation where a patient had been told minutes before surgery that they were ineligible and would have to pay for treatment. A second defined the role as having a *"degree of racial profiling"* due to time constraints and how they suspect or would *"be surprised if they had no role in data sharing…"* **[B04]**, highlighting the perceived link between the two policies.

For peer review only

For peer review only - http://bmjopen.bmj.com/site/about/guidelines.xhtml

DISCUSSION

Key findings

We have shown that there is firstly, a varied understanding of the data sharing agreement among healthcare professionals and volunteers who work closely with migrants, attributed to a lack of transparency in the development and dissemination of the policy by government. Secondly, we heard significant concerns about the impact of the data sharing agreement on migrants' already compromised access to healthcare, and on the relationship between clinicians and patients which is founded on principles of trust and confidentiality.[52] Thirdly, we identified worrying examples of how the data sharing agreement could adversely affect quality and safety of patient care, through patients withholding information or clinicians potentially bypassing standard recordkeeping in an attempt to protect patient confidentiality.

The study resonated with previously reported barriers to migrants accessing health services, including perceived discrimination.[29 33] Participants referred to both healthcare charges and data sharing policies in their narratives, suggesting that they are perceived to be closely related. Findings were influenced by the participant's clinical setting; sexual health providers emphasised confidentiality as a key feature, whereas a psychiatrist discussed how disclosing information was important for managing safety and 'risk'. This highlights an understanding that some data sharing will always be required. However, there is a delicate balance which must be communicated with patients to avoid creating any additional barriers to accessing services. Overall, participants largely disagreed with patient data sharing for immigration purposes and perceived the agreement as instilling fear of deportation, with some alluding to potential future data sharing misuses with other government departments. This echoes Foucault's[53] description of pervasive power associated with surveillance and monitoring evident across society and intersecting with aspects of life including health and wellbeing. Some participants who knew less about the agreement were indecisive about the amendment made for 'serious' crime which may be due to a lack of understanding and insufficient time to consider their view.

Strengths and limitations of the study

There were several strengths to this study, predominantly that to our knowledge, this is the first study to investigate healthcare providers' perspectives of the policy. The interviews generated large amounts of rich, contextual data with a reflexive process acknowledging the active role and influences of the researcher.[45] Coding and themes were discussed (HW, AWS), providing additional methodological rigour. Furthermore, conducting the study as an academic (VP) provided the opportunity to ask challenging questions; for instance, exploring relationships with colleagues, an area overlooked in *a priori* themes. The main study limitation was sample size due to recruitment difficulties including the limited availability of healthcare

For peer review only - http://bmjopen.bmj.com/site/about/guidelines.xhtml

providers and time constraints. However, interviews were conducted until no new codes emerged and inductive thematic saturation was met. Participants were aware of the study purpose which may have influenced recruitment and responses and the non-clinical background of VP will have influenced the analysis and interpretation of findings.

Meaning of the study

Policymakers should consider the relative importance of the implications of patient data sharing agreements to individual and wider public health when monitoring their implementation. For instance, some vulnerable sub-groups, such as pregnant women, were perceived to be negatively affected by the policy. However, these are only the cases that presented to healthcare and there will inevitably be those too afraid to access health services (including undocumented migrants) due to the policy. Secondly, the involvement of medical ethicists, independent advisors and Caldicott Guardians should be considered for future intra-governmental data sharing arrangements that may impact healthcare access.[54] Participants suggested that stakeholder consultations would improve the understanding of governmental decision-making and would support the rebuilding of trust between government, the public and NHS workforce. Finally, health policies should be considered in relation to the NHS principles as there may be consequences to clinical care delivered if these are undermined.

Future work

Future research should explore the impact of data sharing and other policies and contexts on migrant healthcare access and health. Further qualitative work should include all stakeholders including migrants, community organisations, health and social care providers and policymakers, and be linked to quantitative research on service use, and larger surveys to capture concrete examples of issues in quality and safety. Conducting research in areas outside of London that have seen an influx of migration would be an interesting narrative to explore as at least 75% of participants interviewed in the study worked in the diverse capital.

Conclusion

The study raises important and pertinent questions around the role of policies in influencing migrants' access, uptake and presentation to healthcare services. The study findings suggest that this data sharing agreement created tension with the NHS principles. Although some case studies may not be directly linked to the data sharing agreement, they indicated how blurring the lines between healthcare and immigration control can influence care received. As such the formalisation, implementation and amendment of this policy could be defined as a significant example of where perceived lack of transparency can lead to suspicion, distrust

For peer review only - http://bmjopen.bmj.com/site/about/guidelines.xhtml

and fear towards government by both NHS workers and patients and can potentially result in harm to both patient safety and the wider public health.

For peer review only

For peer review only - http://bmjopen.bmj.com/site/about/guidelines.xhtml

Acknowledgements: We would like to thank all the study participants for taking the time to share their views.

Author's contribution: VP, HW, AWS, ICM: study concept and design; VP: conducted, transcribed and analysed all interviews and drafted the manuscript; HW, AWS: supported with analysis of transcripts; HW, AWS, ICM: contributed to critical advice and revisions of the manuscript. All authors read and approved the final manuscript.

Funding statement: This research received no specific grant from any funding agency in the public, commercial or not-for-profit sectors. HW receives support from the National Institute for Health Research (NIHR) Biomedical Research Centre based at Imperial College Healthcare NHS Trust and Imperial College London.

Competing interests: None declared.

Data sharing statement: Audio files were destroyed immediately after transcription with participants designated a non-identifiable code to ensure anonymity. Anonymised transcripts are stored in an encrypted and password-protected environment at Imperial College London. No additional data available.

Transparency statement: The lead author affirms that the manuscript is an honest, accurate and transparent account of the study being reported; and that any discrepancies from the study as originally planned (and, if relevant, registered) have been explained.

Ethical approval: Granted by Imperial College London (Ref: 18IC4526) in April 2018 and Public Health England (Ref: R&D H088) in July 2018, with the ethics board notified of any amendments made. All participants gave written and verbal consent before taking part.

For peer review only

REFERENCES

1. Home Office, Department of Health, NHS Digital. [WITHDRAWN] Memorandum of Understanding between Health and Social Care Information Centre and the Home Office and the Department of Health, 2019.

2. Gulland A. Handing NHS data to the Home Office. *BMJ* 2017;356

3. NHS Digital. Written evidence from NHS Digital (MOU0004): House of Commons, 2018.

4. Public Health England. Data-sharing MoU between NHS Digital and Home Office: call for evidence 2018 [Available from: https://www.gov.uk/government/consultations/data-sharing-mou-between-nhs-digital-and-home-office-call-for-evidence accessed 24 February 2018.

5. Britz JB, McKee M. Charging migrants for health care could compromise public health and increase costs for the NHS. *Journal of Public Health* 2016;38(2):384-90. doi: 10.1093/pubmed/fdv043

6. Dixon S, Agha K, Ali F, et al. Female genital mutilation in the UK- where are we, where do we go next? Involving communities in setting the research agenda. *Research Involvement and Engagement* 2018;4(1):29. doi: 10.1186/s40900-018-0103-5

7. Whelan I. The effect of United Kingdom immigration policies on migrant access to sexual and reproductive healthcare. *BMJ Sexual & Reproductive Health* 2019;45:74-77.

8. Torjesen I. Home Office powers to check NHS immigration data is severely restricted. *BMJ* 2018;361

9. NHS Digital. A statement from NHS Digital on the Memorandum of Understanding with the Home Office 2018 [Available from: https://digital.nhs.uk/news-and-events/latest-news/a-statement-from-nhs-digital-on-the-memorandum-of-understanding-with-the-home-office accessed 11 August 2018.

10. Information Commissioner's Office. Guide to the General Data Protection Regulation (GDPR) 2018 [Available from: https://assets.publishing.service.gov.uk/government/uploads/system/uploads/attachment_data/file/711097/guide-to-the-general-data-protection-regulation-gdpr-1-0.pdf accessed 22 October 2018.

11. NHS Digital. Memorandum of Understanding Revision Plan 2018 [updated 22 October 2018. Available from: https://digital.nhs.uk/services/national-back-office-for-the-personal-demographics-service/memorandum-of-understanding-revision-plan accessed 15 November 2018.

12. Patel A. Government could automatically access GP patient data for benefits assessments: Pulse; 2019 [Available from: http://www.pulsetoday.co.uk/news/gp-topics/legal/government-could-automatically-access-gp-patient-data-for-benefits-assessments/20038333.article accessed 15 May 2019.

13. Digital Marketplace. NHS / DWP data-sharing technical proof of concept: Gov.uk; 2019 [Available from: https://www.digitalmarketplace.service.gov.uk/digital-outcomes-and-specialists/opportunities/8859 accessed 15 May 2019.

14. Cuadra CB. Right of access to health care for undocumented migrants in EU: a comparative study of national policies. *European Journal of Public Health* 2012;22(2):267-71. doi: 10.1093/eurpub/ckr049

For peer review only - http://bmjopen.bmj.com/site/about/guidelines.xhtml

15. Abubakar I, Aldridge RW, Devakumar D, et al. The UCL–Lancet Commission on Migration and Health: the health of a world on the move. *The Lancet* 2018;392(10164):2606-54. doi: https://doi.org/10.1016/S0140-6736(18)32114-7

16. Hannigan A, O'Donnell P, O'Keeffe M, et al. How do variations in definitions of “migrant” and their application influence the access of migrants to health care services? Health Evidence Network Synthesis Report. Copenhagen: WHO Regional Office for Europe 2016:50.

17. Norredam M, Nielsen SS, Krasnik A. Migrants’ utilization of somatic healthcare services in Europe—a systematic review. *European Journal of Public Health* 2010;20(5):555-63. doi: 10.1093/eurpub/ckp195

18. Graetz V, Rechel B, Groot W, et al. Utilization of health care services by migrants in Europe—a systematic literature review. *British Medical Bulletin* 2017;121(1):5-18. doi: 10.1093/bmb/ldw057

19. Muñoz M-A, Pastor E, Pujol J, et al. Primary health care utilization by immigrants as compared to the native population: a multilevel analysis of a large clinical database in Catalonia. *European Journal of General Practice* 2012;18(2):100-06. doi: 10.3109/13814788.2012.656085

20. Jiménez-Rubio D, Hernández-Quevedo C. Inequalities in the use of health services between immigrants and the native population in Spain: what is driving the differences? *The European Journal of Health Economics* 2011;12(1):17-28. doi: 10.1007/s10198-010-0220-z

21. Vargas ED, Sanchez GR, Juárez M. Fear by Association: Perceptions of Anti-Immigrant Policy and Health Outcomes. *Journal of Health Politics, Policy and Law* 2017;42(3):459-83. doi: 10.1215/03616878-3802940

22. Gulbas LE, Zayas LH, Yoon H, et al. Deportation experiences and depression among U.S. citizen-children with undocumented Mexican parents. *Child: Care, Health and Development* 2016;42(2):220-30. doi: doi:10.1111/cch.12307

23. Saadi A, McKee M. Hospitals as places of sanctuary. *BMJ* 2018;361:k2178. doi: 10.1136/bmj.k2178

24. Hacker K, Anies M, Folb BL, et al. Barriers to health care for undocumented immigrants: a literature review. *Risk management and healthcare policy* 2015;8:175-83. doi: 10.2147/RMHP.S70173

25. Juárez SP, Honkaniemi H, Dunlavy AC, et al. Effects of non-health-targeted policies on migrant health: a systematic review and meta-analysis. *The Lancet Global Health* 2019;7(4):e420-e35. doi: 10.1016/S2214-109X(18)30560-6

26. Department of Health and Social Care. Guidance on implementing the overseas visitor charging regulations 2018.

27. Brannan S, Campbell R, Davies M, et al. Ethics briefing: changes to migrant entitlement to free healthcare announced in the UK. *Journal of Medical Ethics* 2014;40(3):213-14. doi: 10.1136/medethics-2014-102061

28. NHS. How to register with a GP practice [updated 13 January 2016. Available from: https://www.nhs.uk/using-the-nhs/nhs-services/gps/how-to-register-with-a-gp-practice/ accessed 12 October 2018.

For peer review only

29. Poduval S, Howard N, Jones L, et al. Experiences Among Undocumented Migrants Accessing Primary Care in the United Kingdom:A Qualitative Study. *International Journal of Health Services* 2015;45(2):320-33. doi: 10.1177/0020731414568511

30. Cooke G, Hargreaves S, Natkunarajah J, et al. Impact on and use of an inner-city London Infectious Diseases Department by international migrants: a questionnaire survey. *BMC Health Services Research* 2007;7(1):113. doi: 10.1186/1472-6963-7-113

31. Davies AR, Chitnis X, Bardsley M. Hospital activity and cost incurred because of unregistered patients in England: considerations for current and new commissioners. *Journal of Public Health* 2013;35(4):590-97. doi: 10.1093/pubmed/fds098

32. Bhatia R, Wallace P. Experiences of refugees and asylum seekers in general practice: a qualitative study. *BMC Family Practice* 2007;8(1):48. doi: 10.1186/1471-2296-8-48

33. O'Donnell CA, Burns N, Mair FS, et al. Reducing the health care burden for marginalised migrants: The potential role for primary care in Europe. *Health Policy* 2016;120(5):495-508. doi: https://doi.org/10.1016/j.healthpol.2016.03.012

34. Aung NC, Rechel B, Odermatt P. Access to and utilisation of GP services among Burmese migrants in London: a cross-sectional descriptive study. *BMC Health Services Research* 2010;10(1):285. doi: 10.1186/1472-6963-10-285

35. Fox H, Hiam L. Migrant access to NHS healthcare. *InnovAiT* 2018;0(0):1755738018774819. doi: 10.1177/1755738018774819

36. Hiam L, Steele S, McKee M. Creating a 'hostile environment for migrants': the British government's use of health service data to restrict immigration is a very bad idea. *Health Econ Policy Law* 2018;13(2):107-17. doi: 10.1017/S1744133117000251

37. Czaika M, De Haas H. Briefing: Determinants of Migration to the UK 2017 [Available from: http://migrationobservatory.ox.ac.uk/wp-content/uploads/2016/04/Briefing-Determinants-of-Migration-17-1.pdf.

38. Liberty (The National Council for Civil Liberties). Liberty's written evidence to the Public Health England consultation on the public health impact of the Memorandum of Understanding on data sharing between NHS Digital, the Home Office, and the Department of Health and Social Care 2018 [Available from: https://www.libertyhumanrights.org.uk/sites/default/files/Libertys%20written%20evidence%20to%20the%20Public%20Health%20England%20consultation%20on%20the%20public%20health%20impact%20of%20the%20MOU%20on%20data%20sharing%20between%20NHSD%20HO%20DHSC_0.pdf accessed 30 May 2019.

39. The United Kingdom Caldicott Guardian Council. [Available from: https://www.ukcgc.uk/ accessed 22 October 2018.

40. Health and Social Care Committee. Memorandum of understanding on datasharing between NHS Digital and the Home Office: Fifth Report of Session 2017–19: House of Commons, 2018:1-33.

41. Bates E. Safe surgeries: how Doctors of the World are helping migrants access healthcare. *BMJ* 2019;364

42. Rafighi E, Poduval S, Legido-Quigley H, et al. National Health Service Principles as Experienced by Vulnerable London Migrants in "Austerity Britain": A Qualitative Study of

For peer review only - http://bmjopen.bmj.com/site/about/guidelines.xhtml

Rights, Entitlements, and Civil-Society Advocacy. *Int J Health Policy Manag* 2016;5(10):589-97. doi: 10.15171/ijhpm.2016.50

43. Saunders B, Sim J, Kingstone T, et al. Saturation in qualitative research: exploring its conceptualization and operationalization. *Quality & Quantity* 2018;52(4):1893-907. doi: 10.1007/s11135-017-0574-8

44. Brinkmann S, Kvale S. InterViews: Learning the Craft of Qualitative Research Interviewing. 3rd ed: SAGE Publications 2015.

45. Charmaz K, Thornberg R, Keane E. Chapter 18: Evolving Grounded Theory and Social Justice Inquiry. In: Denzin NK, Lincoln YS, eds. The SAGE Handbook of Qualitative Research. 5th ed: SAGE Publications 2018:411-43.

46. Glaser BG, Strauss AL. The Discovery of Grounded Theory: Strategies for Qualitative Research: Aldine 1967.

47. Scholl J. Escaping the Conceptual Analysis Straightjacket: Pathological Mechanisms and Canguilhem's Biological Philosophy. *Perspectives in Biology and Medicine* 2015;58(4):395–418.

48. Tong A, Sainsbury P, Craig J. Consolidated criteria for reporting qualitative research (COREQ): a 32-item checklist for interviews and focus groups. *International Journal for Quality in Health Care* 2007;19(6):349-57. doi: 10.1093/intqhc/mzm042

49. Doctors of the World UK. Safe Surgeries Toolkit 2018 [Available from: https://www.doctorsoftheworld.org.uk/safe-surgeries-toolkit accessed 28 May 2018.

50. Health and Social Care Committee. Memorandum of understanding on data-sharing inquiry 2018 [Available from: https://www.parliament.uk/business/committees/committees-a-z/commons-select/health-committee/inquiries/parliament-2017/mou-data-sharing-nhs-digital-home-office-inquiry-17-19/ accessed 20 November 2018.

51. McKee M. Lessons from the Windrush generation. *BMJ* 2018;361

52. General Medical Council. Confidentiality: good practice in handling patient information 2017 [updated 25 May 2018. Available from: https://www.gmc-uk.org/-/media/documents/confidentiality-good-practice-in-handling-patient-information---english-0417_pdf-70080105.pdf accessed 28 May 2018.

53. Foucault M. The Subject and Power. *Critical Inquiry* 1982;8(4):777-95. doi: 10.1086/448181

54. Oral evidence: Memorandum of understanding on data-sharing. Health and Social Care Committee. HC 677 ed: House of Commons., 2018.

For peer review only - http://bmjopen.bmj.com/site/about/guidelines.xhtml

TABLES

Table 1: Summary of study participants' demographics..8

FIGURE LEGENDS

Figure 1: A conceptual thematic framework of key themes and sub-themes identified through data analysis used to organise findings. The framework also explores each themes relationship within the NHS principles and at an individual (micro), organisational (meso) and societal (macro) level ………………………………………...……………..……………...8

Figure 2: The patient pathway for primary care services and the perceived barriers migrant patients may face as described by participants and the literature. Perceived barriers highlighted in **bold** were described during interviews ………………………………………10

For peer review only

For peer review only - http://bmjopen.bmj.com/site/about/guidelines.xhtml

Figure 1: A conceptual thematic framework of key themes and sub-themes identified through data analysis used to organise findings. The framework also explores each themes relationship within the NHS principles and at an individual (micro), organisational (meso) and societal (macro) level.

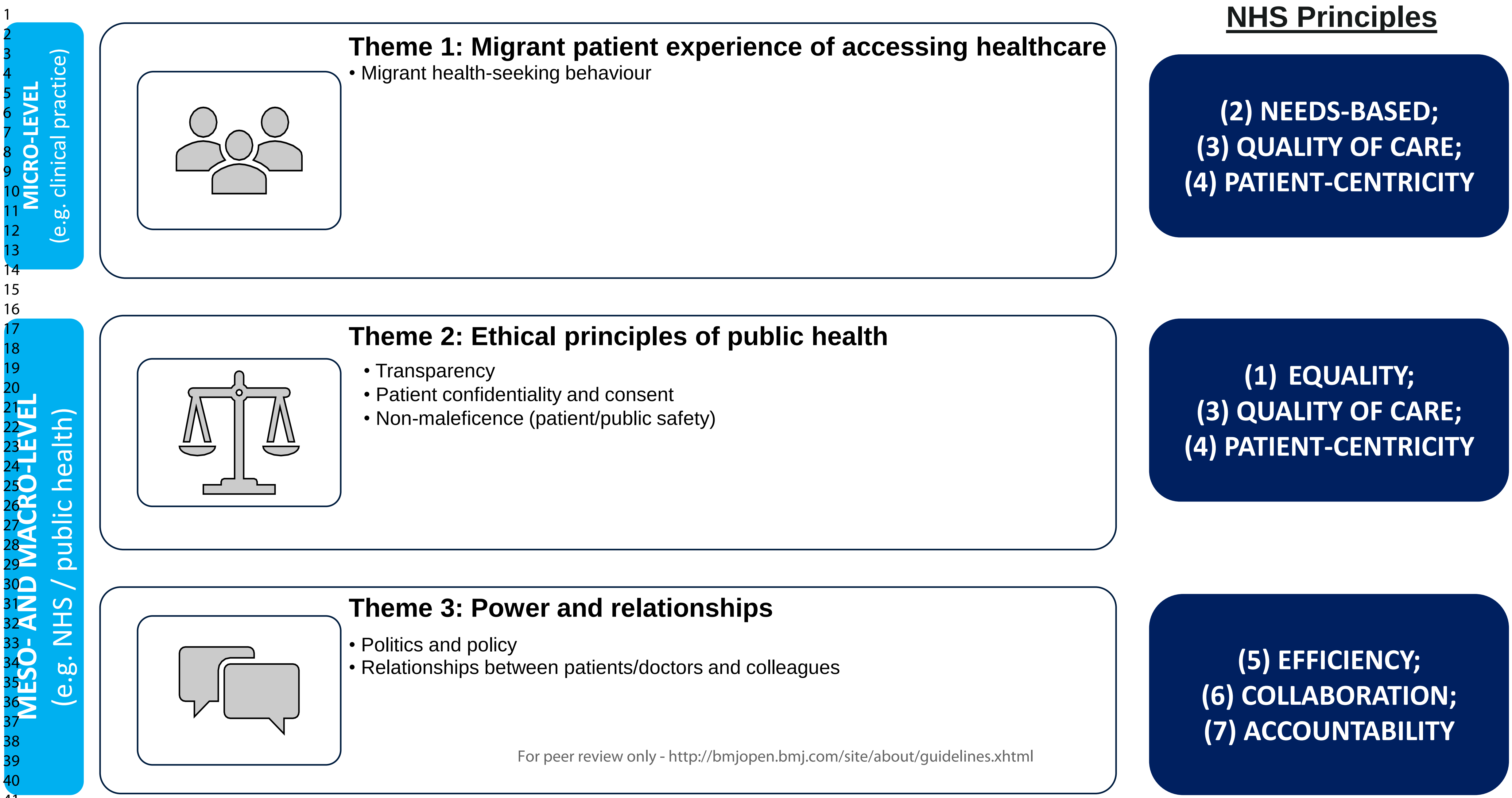

For peer review only - http://bmjopen.bmj.com/site/about/guidelines.xhtml

Figure 2: The patient pathway for primary care services and the perceived barriers migrant patients may face as described by participants and the literature. Perceived barriers highlighted in **bold** were described during interviews.

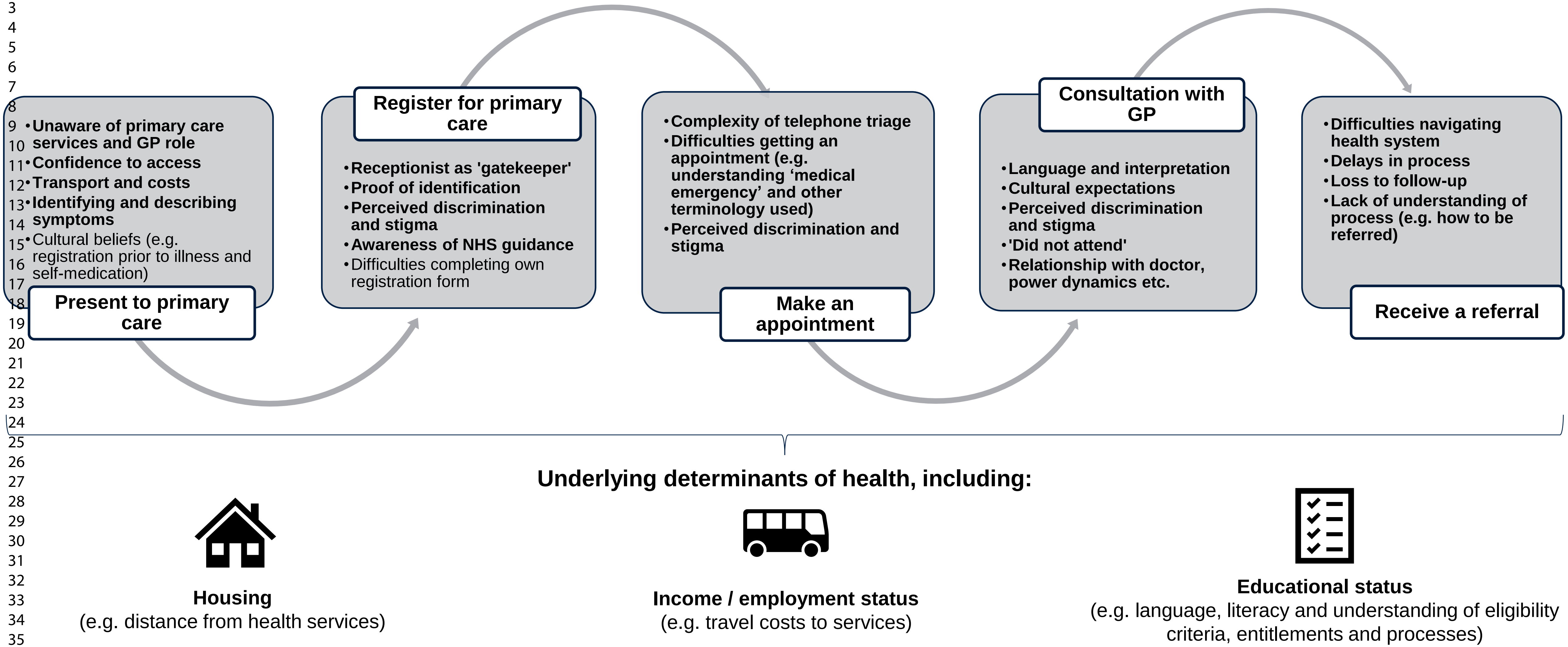

Abbreviations: GP, General Practice or Practitioner; NHS, National Health Service

Supplementary Appendix 1: Non-clinical data shared in accordance to the MoU, including if individual is deceased. Source: NHS Digital.

Non-clinical data shared

- Name (and date last known to be effective)
- Gender
- Date of birth (and date last known to be effective)
- Address
- Primary care support area code
- Primary care support area name
- Date of GP/primary care registration
- Primary care support England contact details (email and telephone number)

Data shared (if deceased)

- Name (and date last known to be effective)
- Date of birth
- Death registration district
- Death registration year/quarter

REFERENCE: NHS Digital. Written evidence from NHS Digital (MOU0004): House of Commons, 2018.

For peer review only

Supplementary Appendix 2: Interview discussion topic guide

Introductory Questions

1. Can you please tell me more about your organisation and your role?

2. How many years have you been working in this role?

3. How many years have you been practicing medicine?

Theme 1: Awareness, understanding, perceptions of patient data sharing, as described in the Home Office, Department of Health, NHS Digital MoU

1. Does your practice /organisation collect demographic data about people who come to access services? If so, please could you tell me the type of data that is collected and recorded? (e.g. name, DOB, country of birth, NHS number, current address (is this mandatory?), telephone number)

2. Outside of the NHS system, who may have access to patient demographic data?

3. Who do you think should have access to patient demographic data, outside of the NHS? Prompts: Why? Why Not?

4. What can you tell me about NHS Digital sharing data with the Home Office?

ONLY *if they* ***do not know anything*** *about it, read out: "Since 2013, NHS Digital had been informally sharing patient administrative data, such as name, date of birth and address with the Home Office on an ad hoc basis to assist the Home Office with locating and prosecuting migrants in relation to immigration issues. That process was formalised in a Memorandum of Understanding in January 2017. This agreement was amended in May 2018. "*

a. How did you hear about this data sharing arrangement?

b. When do you first remember hearing about it?

c. What do you think about this policy? (of NHS Digital sharing demographic data with the Home Office in order for the Home Office to investigate immigration offences; previously any offenders but now only individuals convicted of, or under investigation for, serious crime?)

5. Has your awareness about this data sharing influenced how your practice / organisation records patient administrative data? If so, how?

6. Has knowledge of the data sharing changed the advice you give to patients? If so, how?

7. How was the data sharing viewed by your colleagues? Organisation? and peers?

8. Can you tell me about any interactions that you have had with patients about the data sharing?

9. Based on your experience, did knowledge that administrative data was being shared between NHS Digital and the Home Office have implications for how patients felt about accessing your services? How so?

Theme 2: Experience with migrant patients

For peer review only

10. In your work as XXX (e.g. GP), do you see patients who have been born in a country other than the UK, i.e. migrants?

11. Can you tell me more about this patient population? Prompts: Country of origin, English language ability

12. What have your experiences been like with migrant patients? (i.e. compared to UK born patients)?

13. From your experience, what can you tell me about their uptake of primary health services? Compared to British patients?

14. In your experience, is there a distinct different with migrant patients' health needs?

15. Thinking about your own experience, are you aware of any barriers that migrants might face in accessing services? Can you expand on what these might be and why?

16. From your experience, have migrant patients expressed any concerns about their data being shared with the Home Office, or concerns that accessing health services may lead to immigration authorities contacting them?

17. Thinking about this issue, has the data sharing affected your relationship with migrant patients?

Theme 3: Patient confidentiality

18. As a clinician, how you do view this data sharing in relation to patient confidentiality? Prompts: Why? How?

19. How do you think the NHS Digital data sharing may be perceived by the public? Specifically, for example public trust in NHS' management and guardianship of patient data?

Theme 4: Patient safety

IF participant cites fear of immigration or data sharing as a barrier for migrants' uptake of health services:

20. In your opinion, what are the implications of the data sharing for patient safety? For migrant patients? For vulnerable populations such as pregnant women, children, people with infectious diseases?

21. Lastly, what do you think of the data sharing in the broader terms of the general public health?

Thank you for your time. Do you have any questions for me?

****END RECORDING****

For peer review only

Supplementary Appendix 3: The 7 NHS Principles of the NHS Constitution for England. Source: Department of Health and Social Care.

NHS Principle	Summarised term
1. Provide comprehensive service, available to all	Universal access (equality)
2. Access based on clinical need, not individual's ability to pay	Needs-based
3. Aspires to the highest standards of excellence and professionalism	Quality of care
4. Patient at the heart of everything NHS does	Patient-centricity
5. Working across organisational boundaries	Collaboration
6. Provides best value for taxpayer's money	(Economic) Efficiency
7. Accountable to public, communities and patients it serves	Accountability

REFERENCE: Department of Health and Social Care. *The NHS Constitution for England*. [Updated October 2015] Available from: https://www.gov.uk/government/publications/the-nhsconstitution-for-england/the-nhs-constitution-for-england [Accessed: 17th May 2018].

For peer review only

Supplementary Appendix 4: COREQ (Consolidated criteria for Reporting Qualitative research) Checklist

Manuscript

Patient data sharing for immigration enforcement: a qualitative study of healthcare providers in England

**Topic and item number**	**Guide questions/description**	**Detail/ page number**
**Domain 1: Research team and reflexivity**		
Personal Characteristics		
1. Interviewer/facilitator	Which author/s conducted the interview or focus group?	6
2. Credentials	What were the researcher's credentials? E.g. PhD, MD	BSc
3. Occupation	What was their occupation at the time of the study?	6
4. Gender	Was the researcher male or female?	6
5. Experience and training	What experience or training did the researcher have?	At the time, VP was completing MPH degree and received training in qualitative research methods.
Relationship with participants		
6. Relationship established	Was a relationship established prior to study commencement?	Yes
7. Participant knowledge of the interviewer	What did the participants know about the researcher? e.g. personal goals, reasons for doing the research	6, 15<br><br>Participants were made aware that this formed part of an MPH research project – this was outlined on the participant information sheet (PIS).
8. Interviewer characteristics	What characteristics were reported about the interviewer/facilitator? e.g. Bias, assumptions, reasons and interests in the research topic	6, 7, 15<br><br>VP was a postgraduate student who selected the research project. The project was in collaboration with Public Health England – participants were made aware of this in the PIS.
**Domain 2: study design**		
Theoretical framework		
9. Methodological orientation and Theory	What methodological orientation was stated to underpin the study? e.g. grounded theory, discourse analysis, ethnography, phenomenology, content analysis	6, 7
Participant selection		

Developed from: Tong A, Sainsbury P, Craig J. Consolidated criteria for reporting qualitative research (COREQ): a 32-item checklist for interviews and focus groups. *International Journal for Quality in Health Care*. 2007. Volume 19, Number 6: pp. 349 – 357

For peer review only

10. Sampling	How were participants selected? e.g. purposive, convenience, consecutive, snowball	6
11. Method of approach	How were participants approached? e.g. face-to-face, telephone, mail, email	6
12. Sample size	How many participants were in the study?	6
13. Non-participation	How many people refused to participate or dropped out? Reasons?	N/A – the first 12 participants invited took part and gave informed consent to take part. No participants subsequently refused or dropped out.
Setting		
14. Setting of data collection	Where was the data collected? e.g. home, clinic, workplace	6
15. Presence of non-participants	Was anyone else present besides the participants and researchers?	No
16. Description of sample	What are the important characteristics of the sample? e.g. demographic data, date	8
Data collection		
17. Interview guide	Were questions, prompts, guides provided by the authors? Was it pilot tested?	6, 7
18. Repeat interviews	Were repeat interviews carried out? If yes, how many?	No
19. Audio/visual recording	Did the research use audio or visual recording to collect the data?	7
20. Field notes	Were field notes made during and/or after the interview or focus group?	7
21. Duration	What was the duration of the interviews or focus group?	8
22. Data saturation	Was data saturation discussed?	6, 15
23. Transcripts returned	Were transcripts returned to participants for comment and/or correction?	No
**Domain 3: analysis and findings**		
Data analysis		
24. Number of data coders	How many data coders coded the data?	7, 14
25. Description of the coding tree	Did authors provide a description of the coding tree?	7
26. Derivation of themes	Were themes identified in advance or derived from the data?	6, 7
27. Software	What software, if applicable, was used to manage the data?	7<br>NVivo
28. Participant checking	Did participants provide feedback on the findings?	No
Reporting		
29. Quotations presented	Were participant quotations presented to illustrate the themes/findings? Was each quotation identified? e.g. participant number	9-13
30. Data and findings consistent	Was there consistency between the data presented and the findings?	Yes

Developed from: Tong A, Sainsbury P, Craig J. Consolidated criteria for reporting qualitative research (COREQ): a 32-item checklist for interviews and focus groups. *International Journal for Quality in Health Care*. 2007. Volume 19, Number 6: pp. 349 – 357

For peer review only

31. Clarity of major themes	Were major themes clearly presented in the findings?	Yes
32. Clarity of minor themes	Is there a description of diverse cases or discussion of minor themes?	Yes -subthemes discussed under headings of major themes

For peer review only

Developed from: Tong A, Sainsbury P, Craig J. Consolidated criteria for reporting qualitative research (COREQ): a 32-item checklist for interviews and focus groups. *International Journal for Quality in Health Care*. 2007. Volume 19, Number 6: pp. 349 – 357

BMJ Open

Patient data sharing for immigration enforcement: a qualitative study of healthcare providers in England

Journal:	*BMJ Open*
Manuscript ID	bmjopen-2019-033202.R1
Article Type:	Research
Date Submitted by the Author:	12-Nov-2019
Complete List of Authors:	Papageorgiou, Vasiliki; Imperial College London School of Public Health, Patient Experience Research Centre<br>Wharton-Smith, Alexandra; London School of Hygiene and Tropical Medicine Faculty of Public Health and Policy, Department of Global Health and Development<br>Campos-Matos, Ines; Public Health England, Migration Health, Health Improvement Directorate; University College London Institute of Epidemiology and Health Care<br>Ward, Helen; Imperial College London School of Public Health, Patient Experience Research Centre
**Primary Subject Heading**:	Public health
Secondary Subject Heading:	Health policy, Ethics
Keywords:	Health policy < HEALTH SERVICES ADMINISTRATION & MANAGEMENT, MEDICAL ETHICS, QUALITATIVE RESEARCH, Migrant Health, PUBLIC HEALTH

SCHOLARONE™
Manuscripts

I, the Submitting Author has the right to grant and does grant on behalf of all authors of the Work (as defined in the below author licence), an exclusive licence and/or a non-exclusive licence for contributions from authors who are: i) UK Crown employees; ii) where BMJ has agreed a CC-BY licence shall apply, and/or iii) in accordance with the terms applicable for US Federal Government officers or employees acting as part of their official duties; on a worldwide, perpetual, irrevocable, royalty-free basis to BMJ Publishing Group Ltd ("BMJ") its licensees and where the relevant Journal is co-owned by BMJ to the co-owners of the Journal, to publish the Work in this journal and any other BMJ products and to exploit all rights, as set out in our licence.

The Submitting Author accepts and understands that any supply made under these terms is made by BMJ to the Submitting Author unless you are acting as an employee on behalf of your employer or a postgraduate student of an affiliated institution which is paying any applicable article publishing charge ("APC") for Open Access articles. Where the Submitting Author wishes to make the Work available on an Open Access basis (and intends to pay the relevant APC), the terms of reuse of such Open Access shall be governed by a Creative Commons licence – details of these licences and which Creative Commons licence will apply to this Work are set out in our licence referred to above.

Other than as permitted in any relevant BMJ Author's Self Archiving Policies, I confirm this Work has not been accepted for publication elsewhere, is not being considered for publication elsewhere and does not duplicate material already published. I confirm all authors consent to publication of this Work and authorise the granting of this licence.

For peer review only

Title	**Patient data sharing for immigration enforcement: a qualitative study of healthcare providers in England**
**Author's names**	Vasiliki Papageorgiou (0000-0002-2387-6780), Alexandra Wharton-Smith (0000-0001-5335-2059), Ines Campos-Matos (0000-0002-6480-4310), Helen Ward (0000-0001-8238-5036)
**Address for each author, authors' names and positions**	• Research Assistant, Patient Experience Research Centre, School of Public Health, Imperial College London, UK<br>• PhD Candidate, Department of Global Health and Development, Faculty of Public Health and Policy, London School of Hygiene and Tropical Medicine, UK<br>• Public Health consultant, Migration Health, Health Improvement Directorate, Public Health England, UK and Institute of Epidemiology and Health Care, University College London, UK<br>• Professor of Public Health, Patient Experience Research Centre, School of Public Health, Imperial College London, UK
**Corresponding author**	Correspondence to:<br>Vasiliki Papageorgiou, vasiliki.papageorgiou17@imperial.ac.uk
**Word count**	4537 words

For peer review only - http://bmjopen.bmj.com/site/about/guidelines.xhtml

ABSTRACT

Aim: To explore healthcare providers' perceptions and experiences of the implications of a patient data sharing agreement between NHS Digital and the Home Office on access to NHS services and quality of care received by migrant patients in England.

Design: A qualitative study using semi-structured interviews, thematic analysis and constant-comparison approach.

Participants: 11 healthcare providers and 1 non-clinical volunteer working in community or hospital-based settings who had experience of migrants accessing NHS England services. Interviews were carried out in 2018.

Setting: England.

Results: Awareness and understanding of the patient data sharing agreement varied among participants, who associated this with a perceived lack of transparency by government. Participants provided insight into how they thought the data sharing agreement was negatively influencing migrants' health-seeking behaviour, their relationship with clinicians and the safety and quality of their care. They referred to the policy as a challenge to their core ethical principles, explicitly patient confidentiality and trust, which varied depending on their clinical specialty.

Conclusions: A perceived lack of transparency during the policy development process can result in suspicion or mistrust towards government amongst the health workforce, patients and public, which is underpinned by a notion of power or control. The patient data sharing agreement was considered a threat to some of the core principles of the NHS and its implementation as adversely affecting healthcare access and patient safety. Future policy development should involve a range of stakeholders including civil society, healthcare professionals and ethicists, and include more meaningful assessments of the impact on healthcare and public health.

Keywords: Health Policy; Medical Ethics; Migrant Health; Public Health; Qualitative Research.

For peer review only - http://bmjopen.bmj.com/site/about/guidelines.xhtml

SUMMARY

Strengths and limitations of this study

- To our knowledge, this is the first exploratory study of the experiences and perspectives of healthcare providers in relation to the patient data sharing agreement between NHS Digital and the Home Office.
- The interviews generated rich, contextual data.
- The findings were interpreted using a social constructionist approach, acknowledging the researcher's own biases through a reflexive process and discussed with others to provide additional rigour.
- The main study limitation was sample size due to the limited availability of healthcare providers and difficulties with recruitment.
- Participants were also aware of the study purpose prior to taking part which may have influenced recruitment and the findings.

For peer review only

For peer review only - http://bmjopen.bmj.com/site/about/guidelines.xhtml

INTRODUCTION

In 2017, a Memorandum of Understanding (referred to here as the data sharing agreement) was agreed between the UK Home Office and NHS Digital, with the Department of Health and Social Care acting as a signatory.[1] This formalised an existing arrangement, in place since 2013, whereby non-clinical patient data **(Supplementary 1)** were being shared to trace suspected immigration offenders, without patient consent nor sometimes the knowledge of clinicians; in some cases, primary care practices were asked directly by the Home Office to confirm patients' addresses.[1 2] NHS Digital ruled the process as justified as the information requested excluded clinical data and "lies at the least intrusive end of the spectrum of confidential information".[3] However, concerns were raised by the public health community (including researchers and clinicians) and advocacy groups, about the potential impact of the data sharing agreement on access to NHS services by diverse and often vulnerable migrant populations, and to the wider public health, including sexual and reproductive health.[4-7] Following these objections, an amendment was made by the UK government (in the House of Commons) on 9 May 2018, to restrict requests to individuals being considered for deportation and convicted of 'serious' criminal offences, or presenting a risk to public security, to ensure compliance with the newly introduced EU General Data Protection Regulation (GDPR) on 25 May 2018.[8-12] GDPR was introduced by the European Parliament and Council to provide greater rights and increased transparency to all EU/EEA citizens in the control, management and processing of their 'personal data' by organisations based within, and outside of, the EU.[11 12] The new UK Home Office/NHS Digital data sharing agreement has yet to be published. However, a revision plan has been detailed by NHS Digital and the original agreement has now been withdrawn.[1 13] Recently, proposals for a similar agreement to determine health-related welfare entitlements between the NHS and Department for Work and Pensions, have also been reported.[14 15] A summary of the historical context to the patient data sharing agreement is given in **Figure 1.**

The decision to migrate is often influenced by both internal and external factors; for instance, perceived prosperity leading to voluntary migration or an increased risk of violence due to conflict resulting in forced migration.[16-19] As a result, the diverse migration journey, including life before and after settling in a new place of residence, can have both a negative and positive influence on health and wellbeing outcomes, in the short-, medium- and long-term.[16 20] In addition, various factors have been shown to influence the health-seeking behaviour and access to healthcare services of documented and undocumented migrants, including cultural ideas and expectations, socio-economic conditions, geographical and institutional factors, the extent and nature of which may vary according to contexts.[5 16 21] However, important gaps exist in the literature on migrant health, health-seeking (or avoidance) behaviour and access

For peer review only - http://bmjopen.bmj.com/site/about/guidelines.xhtml

generally; national and international comparisons are challenging due to differing definitions, contexts and limited epidemiological data.[22 23] For instance, research conducted in Spain has found both a higher and lower utilisation of GP services by different groups of migrants, compared to the native population, due to inequities when seeking initial contact.[24-26] Studies globally, including the United States, have demonstrated that perceived fear of deportation may act as a barrier to migrants accessing health services and has been associated with poor health outcomes including mental health.[27-31] To date, most studies exploring healthcare registration, access or utilisation of services by migrants focus on specific subgroups, populations and areas within a country and are either qualitative[32] or observational studies (including cross-sectional or a retrospective cohort).[23 33 34] Their limitations include, findings not being generalisable to other settings, or not reflecting the transient nature of migration as they only provide a 'snapshot' in time or being influenced by attrition bias.

Within England, Britz and McKee[5] have described how healthcare and living condition inequities can result in preventable morbidity and mortality of vulnerable groups, including migrants, resulting in higher healthcare costs. For instance, charging regulations in England are used to determine eligibility for free NHS services with these guidelines being reported as a barrier for migrants accessing healthcare.[5 35 36] Even where services are free, for example in primary care, access may be limited by registration processes and practices which can vary by GP practice.[37] Additionally, documented and undocumented migrants are often unaware of their entitlements and report mixed experiences during registration, including language barriers.[32] Consequently, there are concerns that unregistered individuals are shifting costs to secondary care due to late presentation with greater clinical severity.[38 39] Discontinuity in care, felt hostility or perceived stigma can further increase difficulties whilst navigating the health system; the healthcare system itself may act as a social determinant.[40 41]

The broader, international, political context has begun to shift towards stricter immigration and border control; for instance, restrictive border control introduced by the Trump administration in the United States[42] and limited entitlements to healthcare by undocumented migrants accessing healthcare services across areas of Europe (e.g. Finland, Ireland).[21 43] Additionally, concerns have been reported about the collection of individuals' migration status in healthcare systems globally, particularly regarding the potential misuse of health and identifiable data, including immigration status and address.[16 29] Critics emphasise that patient confidentiality and trust are incompatible with the UK Home Office/NHS Digital data sharing agreement and NHS charging regulations.[5 10 16 29 44 45] These concerns reflect broader political contexts; a "hostile environment" has been explicitly promoted by the UK government in relation to migration, evident in the application of immigration policy to areas including social welfare since 2010.[46]

For peer review only - http://bmjopen.bmj.com/site/about/guidelines.xhtml

[47] For instance, the UK Immigration Act 2016, explicitly describes measures to restrict support given to individuals (and their dependants) with a rejected asylum seeker application.[48 49] This sits uncomfortably with the increased onus on public and organisational bodies to regulate health and social care data following the introduction of GDPR.[12] Specifically, the UK's independent authority (Information Commissioner's Office) and network of UK Caldicott Guardians are responsible for managing data access requests and protecting an individual's confidentiality within NHS organisations and local authorities providing social services.[11 50 51] Additionally, there is limited research, in both a global and UK context, on the potential conflicts between these different parts of government; for example on the impact of sharing healthcare data for the specific purpose of immigration enforcement.[44-46 50 52-54].

Therefore, it is important to understand the perceptions of the UK Home Office/NHS Digital data sharing agreement by UK healthcare workers (including those in primary, secondary and tertiary care) as migrants may access healthcare at each of these services. This study aims to contribute to the growing evidence of predominantly grey literature[53], editorials[46] and patient case studies[44 45 54], by providing robust data on the experiences of the data sharing agreement among healthcare providers and voluntary sector workers in England. Our aim was to explore whether the policy has influenced clinical care provided and healthcare received by migrant patients and possibly decision-making processes in clinical care and healthcare data management, particularly as a new agreement is currently being drafted. To our knowledge, no previous primary research has been published focussing on this topic among UK healthcare workers.

For peer review only

METHODS

Study design

We carried out a qualitative study using interviews with healthcare providers and a voluntary sector worker. The study was underpinned by a social constructionist approach to explore the connection, power and inequality between the 'macro' (e.g. public health), 'meso' (e.g. Home Office and NHS Digital) and 'micro' levels (e.g. clinical practice), which were contextualised by individual's responses and recognised the interviewer's own biases.[55 56] We drew on the NHS principles of the NHS Constitution for England as a framework, as previously performed by Rafighi *et al*[57], to inform interviewees' perceptions and experience of migrants' access to health services and migrants' health-seeking behaviour.

Recruitment and sampling

Participants were 11 healthcare providers (10 medical doctors, one physiotherapist) and 1 non-clinical volunteer in urban areas of England with experience supporting migrants accessing healthcare services.

Initially, our sampling strategy was to recruit healthcare providers based at NHS sites with large migrant populations (e.g. London). We collected broad geographical location data from study participants and so we are unable to comment on the level of migration at participant locations. To comply with University ethics, we were unable to directly recruit individuals from NHS sites; therefore, participants were recruited from professional bodies, professional networks, social media and existing personal contacts (VP, HW, ICM, AWS) by written email invitation.

Selection initially focused on primary healthcare providers and voluntary sector workers, but we expanded to include healthcare professionals in secondary care following slow initial recruitment due to unavailability and delayed response.

Participants were sent a participant information sheet (PIS) and consent form prior to the interview taking place and given the opportunity to ask any questions about the study.

Data collection

A member of the research team (VP) conducted semi-structured, in-depth interviews in July 2018, either in person (n=7) at the participant's workplace (non-NHS Trust site) or the university campus, or by telephone/online (n=5). Recruitment was stopped at 12 participants due to time constraints of the study. Despite this, we began to see repetition of themes during the interviews.

For peer review only - http://bmjopen.bmj.com/site/about/guidelines.xhtml

An initial topic guide was used to encourage participants to describe their experiences and views of migrants accessing healthcare, both generally and in relation to the data sharing agreement. The interview focussed on the 'how', rather than the 'what', in line with the constructionist concept of this interaction.[58] Some themes were determined *a priori*, with emergent themes explored through subsequent interviews and the relationships between concepts investigated. Four initial themes were explored in the topic guide **(Supplementary 2)** and pilot tested with HW and AWS. If participants were unaware or indicated little understanding of the patient data sharing agreement, a short description was given. Probes were used to encourage participants to elaborate their answers and descriptive field notes were recorded by VP. All interviews were audio recorded and transcribed verbatim by VP.

Data analysis

Memos were written on transcripts by VP describing initial reflections, with the findings analysed using an inductive-deductive approach through constant comparison, derived from grounded theory.[55 59] NVivo 12 was used to organise the data, with a primary thematic framework provided by the topic guide. Coding was completed by VP through initial line-by-line content analysis with subsequent focussed coding and constant-comparison between codes to generate emergent themes and sub-themes.[55] HW and AWS provided consensus on coding by checking the coding framework of the first three transcripts and were approached by VP to check any interpretations made on subsequent transcripts.

A thematic analysis, featuring summarised NHS principles **(Supplementary 3)**, was used to organise data, where appropriate. The framework was not applied to directly categorise data as this would restrict interpretation and overlook the philosophical and empirical concepts being determined.[60]

The findings were shared with Public Health England (PHE) to contribute to their review[4] commissioned by the Department of Health and Social Care. They were also shared with all participants as a lay summary by email. The standards for reporting qualitative research (SRQR) checklist has been used to report study methods and findings **(Supplementary 4)**.[61]

Patient and public involvement

There were no funds or time allocated for public involvement due to the timing of this postgraduate research project.

Ethical approval

For peer review only - http://bmjopen.bmj.com/site/about/guidelines.xhtml

Participants received a participant information sheet and provided written and oral consent. Ethical approval was obtained from Imperial College London (18IC4526) and Public Health England (R&D H088).

For peer review only

For peer review only - http://bmjopen.bmj.com/site/about/guidelines.xhtml

RESULTS

Study population/characteristics

Twelve participants were interviewed, with most based in London **(Table 1)**. Interviews lasted, on average, for 40 minutes (range 20-60). Participants worked in a range of specialties: general practice (n=4), infectious diseases and/or sexual health (n=4), psychiatry (n=1), physiotherapy (n=1), paediatrics (n=1) and community engagement (n=1). Interviewees included an equal split of men and women and a range of ages and levels of experience.

Table 1: Summary of study participants' demographics

Participant ID	Age (years)	Gender	Clinical setting*	Country of birth	Location of workplace
**B01**	25-34	F	Community	UK	London
**B02**	45-54	F	Community	UK	Other
**B03**	35-44	M	Hospital	Non-UK	London
**B04**	25-34	M	Hospital	UK	London
**B05**	45-54	M	Hospital	UK	London
**B06**	25-34	M	Hospital	Non-UK	Other
**B07**	35-44	M	Community	UK	London
**B08**	55-64	M	Community	UK	London
**B09**	45-54	F	Community	Non-UK	London
**B10**	35-44	F	Community	Non-UK	London
**B11†**	65+	F	N/A – voluntary sector	-	-
**B12**	25-34	F	Hospital	UK	London

Interviewees' knowledge about the data sharing agreement and their understanding varied (4 provided a full description, 5 little description and 3 provided no description). Overall, most participants were confused about the specifics of the policy. Participants expressing some understanding often alluded to having learnt about the agreement either through the media or from colleagues making any subsequent answers an immediate reflection, rather than a considered opinion.

Views on the actual and potential impact of the agreement were embedded in a broader understanding of the challenges facing migrants accessing healthcare and are described here as 3 themes **(Figure 2)**: (1) patient experience, (2) ethical principles of public health and (3) power and relationships. These encompassed the themes explored in the topic guide. The themes interconnected with perceived consequences of the agreement and followed the natural sequence of the patient pathway from accessing to receiving healthcare in the NHS.

Abbreviations: F, Female; M, Male; N/A, Not Applicable; UK, United Kingdom.
* Community (self-referral, primary care and sexual health services) or hospital settings.
† Incomplete data received.

For peer review only - http://bmjopen.bmj.com/site/about/guidelines.xhtml

This included reflections on any changes to clinical practice delivered and the patient-healthcare professional relationship.

Theme 1: Migrant Patient Experience of Accessing Healthcare

Healthcare providers described their interactions with migrant patients along the care pathway, although confusion about the asylum process, healthcare entitlements and definition of the word "migrant" were often described, either as a reflection of their own, or colleagues', practice.

Migrant health-seeking behaviour

Participants described a range of influences on migrants' health-seeking behaviour, with some influences relating directly to enforcement and data sharing and others concerning broader aspects. More than half of participants described discriminatory practices in relation to proof of eligibility:

> *"…informally we sort of found out the people with 'foreign-sounding' names were being targeted for these letters [from NHS Spine portal to confirm their home address if they had not used an NHS service in 6-months]."* **[B02]**

This linked to suggestions that migrants would experience fear and anxiety around registration and accessing health services. Specific references were made to immigration control and patients' fears of arrest or deportation of either themselves or others in their immediate networks by the Home Office:

> *"…I think there is a group of people that are afraid and think that by coming into contact with services, they could […] put themselves on the radar."* **[B05]**

Participants talked about how these resultant fears of accessing health services could be particularly harmful for vulnerable people, for example those affected by mental illness. This reflects the backgrounds and interests of some interviewees; one described a suicidal UK citizen detained under the Mental Health Act whose husband was at risk of deportation and had received a letter from the Home Office:

> *"…we continuously sort of had to reaffirm the limits of the information that we share and how much of it is strictly confidential...."* **[B06]**

For peer review only - http://bmjopen.bmj.com/site/about/guidelines.xhtml

Other perceived influences on health-seeking behaviour included: attitudes to health, cultural ideas and expectations of healthcare services. Participants described how these may result in either delayed presentation or the overuse of healthcare, which could influence public health funding and access to healthcare. For instance, participants working in sexual health and infectious disease services described how delayed or deferred clinical presentation could shift expenses from primary to secondary care:

> *"…if you delay somebody coming forward for HIV care, they're more likely to end up needing an in-patient admission which is more expensive…"* **[B07]**

Figure 3 shows the patient pathway and potential barriers migrants may face when accessing healthcare, which are also influenced by wider determinants. These were identified from the literature and further explored, in relation to the data sharing agreement, during interviews.

Theme 2: Ethical Principles of Public Health

Transparency

Most participants described migrant patients and colleagues as being unaware and having a limited understanding of the policy. They linked this to a "sneaky" agenda, or evasion of transparency by the Home Office to access data without patients' nor clinicians' knowledge:

> *"…GPs have no idea that this precious data they hold is being accessed, sort of on the sly, without them even realising…"* **[B01]**

Concerns were also raised about how patients first hear about the agreement which could generate fear. A non-clinical specialist working for a community-based organisation supporting marginalised populations offered insight into knowledge exchange by rumours, which may influence clinical presentation:

> *"…they will go and share that with all their friends, family […] and they will panic."* **[B11]**

Patient confidentiality and consent

Participants felt that the data sharing agreement directly contradicted the ethical principles of confidentiality and consent that underpinned the professional guidelines they abided by, resulting in an "erosion in trust in healthcare" **[B03].** Specifically, participants working in sexual health described the importance of ensuring confidentiality to individuals accessing these

For peer review only - http://bmjopen.bmj.com/site/about/guidelines.xhtml

services whereas the participant working in mental health services described a difficult balance between managing 'risk' and confidentiality.

One question asked for participants' opinion of the policy in relation to patient confidentiality; four described practices they had taken or were prepared to take to protect confidentiality, which was in line with advice given by Doctors of the World[62] such as changes in recording patient data by keeping separate notes, or writing in shorthand:

> *"…then you'd be thinking […] 'I better not put that [telephone] number in the actual [notes], I'm gonna keep that'…Maybe it's over the top but it does create this kind of space for suspicion."* **[B04]**

For peer review only

Non-maleficence (patient and public safety)

Some participants suggested how migrant patients may bypass the agreement by giving false information or pretending to be someone else, which could impact both individual patient safety and that of the wider public. **B04** describes a case shared by a colleague where this had occurred, and its implications:

> *"…there was a pregnant woman who came to deliver her baby and she was so worried about it she […] used her sister's details... The time that they knew that, was the time they came to give her a blood transfusion, and they realised that her blood group was different…"* **[B04]**

The concept of safety was further explained from the perspective of health protection and public security. Participants reflected on the public safety rationale of the policy, following the recent amendment, offering differing views, with some being less definitive:

> *"From an infectious disease, public health prevention point of view, 'are the people that are likely to have like transmissible illnesses more likely to be the type of people that have committed serious offences?' No…"* **[B03]**

One hospital-based participant spoke of 'security' in terms of the threat of terrorism, from the perspective of having witnessed a terrorist attack, and appeared unsure, *"…I think as far as security goes… I don't know is the answer."* **[B12]**

Participants were able to appreciate "*exceptional circumstances*" **[B02]** where confidentiality may be breached, including criminal investigations or where patients may be at risk of harm to themselves or others, which some described as a "rarity" in their own experience. However,

most participants disagreed with the use of the agreement for immigration purposes, often referring to the ‘philosophy’ or principles of the NHS or medical profession.

Theme 3: Power and relationships

Politics and policy

Participants described the political climate, without probing, referring variously to the “hostile environment”, Brexit, healthcare charges or other policies they viewed as deterring migrants from accessing healthcare. One community clinician described migration as a political issue associated with “scaremongering” by the media, and some participants suggested the need for governmental accountability and transparency. They proposed conducting an informed public debate on the agreement, indicating a lack of awareness of the ‘open’ status of the Health and Social Care Committee inquiry.[63]

Most interviewees mentioned a loss of faith in governmental decision-making processes, specifically the Home Office, following recent media attention on data sharing and immigration scandals, predominantly Windrush. The 2018 Windrush scandal refers to the generation of Caribbean-born British citizens who entered the UK over four decades ago, some of whom were wrongly deported and denied re-entry.[16 64] Some participants also raised concerns of the potential misuse of the agreement by other government agencies, for instance during benefit fraud investigations.

Relationships between patient/healthcare professionals and colleagues

Participants described trust, empathy and rapport as essential elements to the patient-healthcare professional relationship, which they perceived to be challenged by the agreement. Their narrative of the importance of this relationship were described with patients generally:

> *“…if you’ve got, going to have a working relationship with somebody about the most intimate details of their lives […] you have to have that trust.”* **[B02]**

Participants described the positions of other colleagues on the data sharing agreement. One expressed frustration at their local Caldicott Guardian, the person with responsibility for protecting patient confidentiality and compliance with data protection regulations. When approached for guidance on the agreement within their Trust, the Caldicott Guardian had sent:

> *“…a very bland reply saying well, ‘if this is what the government want to do, and they’ve agreed, then we’ll just do it’.”* **[B02]**

For peer review only - http://bmjopen.bmj.com/site/about/guidelines.xhtml

Conversely, **B08**, themselves a Caldicott Guardian, gave an opinion on the legality of the policy, and bemoaned lack of awareness amongst colleagues:

> *"… I believe that* **[thumps table]** *what happened here, did not follow the processes which I certainly thought was there and then, as Caldicott Guardian, thought I was part of."* **[B08]**

Participants also referred to tension between colleagues because of charging policies for overseas visitors. Two participants based in a hospital setting highlighted their negative experience of how overseas visitor officers sought to recover charges from patients. The first clinician described a situation where a patient had been told minutes before surgery that they were ineligible and would have to pay for treatment. A second defined the role as having a *"degree of racial profiling"* due to time constraints and how they "suspect" or would *"be surprised if they had no role in data sharing…"* **[B04]**, highlighting the perceived link between the two policies.

For peer review only

DISCUSSION

Key findings

We have shown that there is firstly, a varied understanding of the data sharing agreement among healthcare professionals and volunteers who work closely with migrants, attributed to a lack of transparency in the development and dissemination of the policy by government. Secondly, we heard significant concerns about the impact of the data sharing agreement on migrants' already compromised access to healthcare, and on the relationship between clinicians and patients which is founded on principles of trust and confidentiality.[65] Thirdly, we identified worrying examples of how the data sharing agreement could adversely affect quality and safety of patient care, through patients withholding information or clinicians potentially bypassing standard recordkeeping in an attempt to protect patient confidentiality.

The study resonated with previously reported barriers to migrants accessing health services, including perceived discrimination.[32 41] Participants referred to both healthcare charges and data sharing policies in their narratives, suggesting that they are perceived to be closely related. Findings were influenced by the participant's clinical setting; sexual health providers emphasised confidentiality as a key feature, whereas a psychiatrist discussed how disclosing information was important for managing safety and 'risk'. This highlights an understanding that some data sharing will always be required. However, there is a delicate balance which must be communicated with patients to avoid creating any additional barriers to accessing services. Overall, participants largely disagreed with patient data sharing for immigration purposes and perceived the agreement as instilling fear of deportation, with some alluding to potential future data sharing misuses with other government departments. This echoes Foucault's[66] description of pervasive power associated with surveillance and monitoring evident across society and intersecting with aspects of life including health and wellbeing. Some participants who knew less about the agreement were indecisive about the amendment made for 'serious' crime which may be due to a lack of understanding and insufficient time to consider their view. However, this also allowed the interviewer to explore the participants' immediate reactions, concerns and responses of sharing non-clinical patient data for immigration purposes.

Strengths and limitations of the study

There were several strengths to this study. The interviews generated rich, contextual data with a reflexive process acknowledging the active role and influences of the researcher.[55] Coding and themes were discussed with authors experienced in healthcare for marginalised groups (HW) and public health research (HW, AWS), providing additional methodological rigour. Furthermore, conducting the study as an academic (VP) provided the opportunity to ask challenging questions; for instance, exploring relationships with colleagues, an area

For peer review only - http://bmjopen.bmj.com/site/about/guidelines.xhtml

overlooked in *a priori* themes. The main study limitation was sample size due to recruitment difficulties including the duration of the study and limited availability of healthcare providers. Further work is required to assess how common these experiences are by healthcare workers working in other clinical settings and geographical regions of the UK. It is likely that recruitment was influenced by participants being aware of the study purpose which, in turn, may have influenced their responses. Additionally, the non-clinical background of VP will have influenced the analysis and interpretation of findings.

Meaning of the study

Policymakers should consider the implications of patient data sharing agreements for individual and wider public health when monitoring their implementation. For instance, some vulnerable sub-groups, such as pregnant women, were perceived to be negatively affected by the policy. However, these are only the cases that presented to healthcare and there will inevitably be those too afraid to access health services (including undocumented migrants) due to the policy. Secondly, the involvement of medical ethicists, independent advisors and Caldicott Guardians should be considered for future intra-governmental data sharing arrangements that may impact healthcare access.[67] Participants suggested that stakeholder consultations would improve the understanding of governmental decision-making and would support the rebuilding of trust between government, the public and NHS workforce. Finally, health policies should be considered in relation to the NHS principles as there may be consequences to clinical care delivered if these are undermined.

Future work

Future research should explore the impact of data sharing and other policies and contexts on migrant healthcare access and health. Further qualitative work should include all stakeholders including migrants, community organisations, health and social care providers and policymakers, and be linked to quantitative research on service use, and larger surveys to capture concrete examples of issues in quality and safety. Conducting research in areas outside of London that have seen an influx of migration would be an interesting narrative to explore as at least 75% of participants interviewed in the study worked in the diverse capital.

Conclusion

The study raises important and pertinent questions around the role of policies in influencing migrants' access, uptake and presentation to healthcare services. The study findings suggest that this data sharing agreement created tension with the NHS principles. Although some case studies may not be directly linked to the data sharing agreement, they indicated how blurring the lines between healthcare and immigration control can influence care received. As such,

For peer review only - http://bmjopen.bmj.com/site/about/guidelines.xhtml

the formalisation, implementation and amendment of this policy could be defined as a significant example of where perceived lack of transparency can lead to suspicion, distrust and fear towards government by both NHS workers and patients and can potentially result in harm to both patient safety and the wider public health.

List of recommendations

- Policymakers monitoring the implementation of patient data sharing agreements should consider how these may have implications to individual and the wider public health, including vulnerable individuals who may no longer present to healthcare services.
- Medical ethicists, independent advisors and Caldicott Guardians should be involved during the formalisation of future UK intra-governmental data sharing agreements that may impact healthcare access.
- UK health policies should be considered in relation to the NHS Principles in order to circumvent any potential consequences to the quality, and safety, of clinical care delivered.

For peer review only

For peer review only - http://bmjopen.bmj.com/site/about/guidelines.xhtml

Acknowledgements: We would like to thank all the study participants for taking the time to share their views.

Author's contribution: VP, HW, AWS, ICM: study concept and design; VP: conducted, transcribed and analysed all interviews and drafted the manuscript; HW, AWS: supported with analysis of transcripts; HW, AWS, ICM: contributed to critical advice and revisions of the manuscript. All authors read and approved the final manuscript.

Funding statement: The research was supported by the National Institute for Health Research (NIHR) Biomedical Research Centre based at Imperial College Healthcare NHS Trust and Imperial College London. The views expressed are those of the author(s) and not necessarily those of the NIHR, Public Health England or the Department of Health and Social Care.

Competing interests: None declared.

Data sharing statement: Audio files were destroyed immediately after transcription with participants designated a non-identifiable code to ensure anonymity. Anonymised transcripts are stored in an encrypted and password-protected environment at Imperial College London. No additional data available.

Transparency statement: The lead author affirms that the manuscript is an honest, accurate and transparent account of the study being reported; and that any discrepancies from the study as originally planned (and, if relevant, registered) have been explained.

Ethical approval: Granted by Imperial College London (Ref: 18IC4526) in April 2018 and Public Health England (Ref: R&D H088) in July 2018, with the ethics board notified of any amendments made. All participants gave written and verbal consent before taking part.

For peer review only

REFERENCES

1. Home Office, Department of Health, NHS Digital. [WITHDRAWN] Memorandum of Understanding between Health and Social Care Information Centre and the Home Office and the Department of Health, 2019.

2. Gulland A. Handing NHS data to the Home Office. *BMJ* 2017;356

3. NHS Digital. Written evidence from NHS Digital (MOU0004): House of Commons, 2018.

4. Public Health England. Data-sharing MoU between NHS Digital and Home Office: call for evidence 2018 [Available from: https://www.gov.uk/government/consultations/data-sharing-mou-between-nhs-digital-and-home-office-call-for-evidence accessed 24 February 2018.

5. Britz JB, McKee M. Charging migrants for health care could compromise public health and increase costs for the NHS. *Journal of Public Health* 2016;38(2):384-90. doi: 10.1093/pubmed/fdv043

6. Dixon S, Agha K, Ali F, et al. Female genital mutilation in the UK- where are we, where do we go next? Involving communities in setting the research agenda. *Research Involvement and Engagement* 2018;4(1):29. doi: 10.1186/s40900-018-0103-5

7. Whelan I. The effect of United Kingdom immigration policies on migrant access to sexual and reproductive healthcare. *BMJ Sexual & Reproductive Health* 2019;45:74-77.

8. Torjesen I. Home Office powers to check NHS immigration data is severely restricted. *BMJ* 2018;361

9. Data Protection Bill [Lords]. House of Commons Hansard. London, 2018.

10. NHS Digital. A statement from NHS Digital on the Memorandum of Understanding with the Home Office 2018 [Available from: https://digital.nhs.uk/news-and-events/latest-news/a-statement-from-nhs-digital-on-the-memorandum-of-understanding-with-the-home-office accessed 11 August 2018.

11. Information Commissioner's Office. Guide to the General Data Protection Regulation (GDPR) 2018 [Available from: https://assets.publishing.service.gov.uk/government/uploads/system/uploads/attachment_data/file/711097/guide-to-the-general-data-protection-regulation-gdpr-1-0.pdf accessed 22 October 2018.

12. EU General Data Protection Regulation (GDPR). Regulation (EU) 2016/679 of the European Parliament and of the Council of 27 April 2016 on the protection of natural persons with regard to the processing of personal data and on the free movement of such data, and repealing Directive 95/46/EC (General Data Protection Regulation). OJ 2016 L 119/1.

13. NHS Digital. Memorandum of Understanding Revision Plan 2018 [updated 22 October 2018. Available from: https://digital.nhs.uk/services/national-back-office-for-the-personal-demographics-service/memorandum-of-understanding-revision-plan accessed 15 November 2018.

14. Patel A. Government could automatically access GP patient data for benefits assessments: Pulse; 2019 [Available from: http://www.pulsetoday.co.uk/news/gp-topics/legal/government-could-automatically-access-gp-patient-data-for-benefits-assessments/20038333.article accessed 15 May 2019.

For peer review only - http://bmjopen.bmj.com/site/about/guidelines.xhtml

15. Digital Marketplace. NHS / DWP data-sharing technical proof of concept: Gov.uk; 2019 [Available from: https://www.digitalmarketplace.service.gov.uk/digital-outcomes-and-specialists/opportunities/8859 accessed 15 May 2019.

16. Abubakar I, Aldridge RW, Devakumar D, et al. The UCL–Lancet Commission on Migration and Health: the health of a world on the move. *The Lancet* 2018;392(10164):2606-54. doi: https://doi.org/10.1016/S0140-6736(18)32114-7

17. Rechel B, Mladovsky P, Ingleby D, et al. Migration and health in an increasingly diverse Europe. *The Lancet* 2013;381(9873):1235-45. doi: 10.1016/S0140-6736(12)62086-8

18. Gushulak BD, MacPherson DW. The basic principles of migration health: Population mobility and gaps in disease prevalence. *Emerging Themes in Epidemiology* 2006;3(1):3. doi: 10.1186/1742-7622-3-3

19. Robertshaw L, Dhesi S, Jones LL. Challenges and facilitators for health professionals providing primary healthcare for refugees and asylum seekers in high-income countries: a systematic review and thematic synthesis of qualitative research. *BMJ Open* 2017;7(8):e015981. doi: 10.1136/bmjopen-2017-015981

20. Zimmerman C, Kiss L, Hossain M. Migration and Health: A Framework for 21st Century Policy-Making. *PLOS Medicine* 2011;8(5):e1001034. doi: 10.1371/journal.pmed.1001034

21. Cuadra CB. Right of access to health care for undocumented migrants in EU: a comparative study of national policies. *European Journal of Public Health* 2012;22(2):267-71. doi: 10.1093/eurpub/ckr049

22. Hannigan A, O'Donnell P, O'Keeffe M, et al. How do variations in definitions of "migrant" and their application influence the access of migrants to health care services? Health Evidence Network Synthesis Report. Copenhagen: WHO Regional Office for Europe 2016:50.

23. Norredam M, Nielsen SS, Krasnik A. Migrants' utilization of somatic healthcare services in Europe—a systematic review. *European Journal of Public Health* 2010;20(5):555-63. doi: 10.1093/eurpub/ckp195

24. Graetz V, Rechel B, Groot W, et al. Utilization of health care services by migrants in Europe—a systematic literature review. *British Medical Bulletin* 2017;121(1):5-18. doi: 10.1093/bmb/ldw057

25. Muñoz M-A, Pastor E, Pujol J, et al. Primary health care utilization by immigrants as compared to the native population: a multilevel analysis of a large clinical database in Catalonia. *European Journal of General Practice* 2012;18(2):100-06. doi: 10.3109/13814788.2012.656085

26. Jiménez-Rubio D, Hernández-Quevedo C. Inequalities in the use of health services between immigrants and the native population in Spain: what is driving the differences? *The European Journal of Health Economics* 2011;12(1):17-28. doi: 10.1007/s10198-010-0220-z

27. Vargas ED, Sanchez GR, Juárez M. Fear by Association: Perceptions of Anti-Immigrant Policy and Health Outcomes. *Journal of Health Politics, Policy and Law* 2017;42(3):459-83. doi: 10.1215/03616878-3802940

28. Gulbas LE, Zayas LH, Yoon H, et al. Deportation experiences and depression among U.S. citizen-children with undocumented Mexican parents. *Child: Care, Health and Development* 2016;42(2):220-30. doi: doi:10.1111/cch.12307

For peer review only

29. Saadi A, McKee M. Hospitals as places of sanctuary. *BMJ* 2018;361:k2178. doi: 10.1136/bmj.k2178

30. Hacker K, Anies M, Folb BL, et al. Barriers to health care for undocumented immigrants: a literature review. *Risk management and healthcare policy* 2015;8:175-83. doi: 10.2147/RMHP.S70173

31. Juárez SP, Honkaniemi H, Dunlavy AC, et al. Effects of non-health-targeted policies on migrant health: a systematic review and meta-analysis. *The Lancet Global Health* 2019;7(4):e420-e35. doi: 10.1016/S2214-109X(18)30560-6

32. Poduval S, Howard N, Jones L, et al. Experiences Among Undocumented Migrants Accessing Primary Care in the United Kingdom: A Qualitative Study. *International Journal of Health Services* 2015;45(2):320-33. doi: 10.1177/0020731414568511

33. Aung NC, Rechel B, Odermatt P. Access to and utilisation of GP services among Burmese migrants in London: a cross-sectional descriptive study. *BMC Health Services Research* 2010;10(1):285. doi: 10.1186/1472-6963-10-285

34. Stagg HR, Jones J, Bickler G, et al. Poor uptake of primary healthcare registration among recent entrants to the UK: a retrospective cohort study. *BMJ Open* 2012;2(4):e001453. doi: 10.1136/bmjopen-2012-001453

35. Department of Health and Social Care. Guidance on implementing the overseas visitor charging regulations 2018.

36. Brannan S, Campbell R, Davies M, et al. Ethics briefing: changes to migrant entitlement to free healthcare announced in the UK. *Journal of Medical Ethics* 2014;40(3):213-14. doi: 10.1136/medethics-2014-102061

37. NHS. How to register with a GP practice [updated 13 January 2016. Available from: https://www.nhs.uk/using-the-nhs/nhs-services/gps/how-to-register-with-a-gp-practice/ accessed 12 October 2018.

38. Cooke G, Hargreaves S, Natkunarajah J, et al. Impact on and use of an inner-city London Infectious Diseases Department by international migrants: a questionnaire survey. *BMC Health Services Research* 2007;7(1):113. doi: 10.1186/1472-6963-7-113

39. Davies AR, Chitnis X, Bardsley M. Hospital activity and cost incurred because of unregistered patients in England: considerations for current and new commissioners. *Journal of Public Health* 2013;35(4):590-97. doi: 10.1093/pubmed/fds098

40. Bhatia R, Wallace P. Experiences of refugees and asylum seekers in general practice: a qualitative study. *BMC Family Practice* 2007;8(1):48. doi: 10.1186/1471-2296-8-48

41. O'Donnell CA, Burns N, Mair FS, et al. Reducing the health care burden for marginalised migrants: The potential role for primary care in Europe. *Health Policy* 2016;120(5):495-508. doi: https://doi.org/10.1016/j.healthpol.2016.03.012

42. Khullar D, Chokshi DA. Challenges for immigrant health in the USA-the road to crisis. *The Lancet* 2019;393(10186):2168-74. doi: 10.1016/S0140-6736(19)30035-2

43. Winters M, Rechel B, de Jong L, et al. A systematic review on the use of healthcare services by undocumented migrants in Europe. *BMC Health Services Research* 2018;18(1):30. doi: 10.1186/s12913-018-2838-y

For peer review only - http://bmjopen.bmj.com/site/about/guidelines.xhtml

44. Fox H, Hiam L. Migrant access to NHS healthcare. *InnovAiT* 2018;0(0):1755738018774819. doi: 10.1177/1755738018774819

45. Reynolds JMK, Mitchell C. 'Inglan is a bitch': hostile NHS charging regulations contravene the ethical principles of the medical profession. *Journal of Medical Ethics* 2019;45(8):497-503. doi: 10.1136/medethics-2019-105419

46. Hiam L, Steele S, McKee M. Creating a 'hostile environment for migrants': the British government's use of health service data to restrict immigration is a very bad idea. *Health Econ Policy Law* 2018;13(2):107-17. doi: 10.1017/S1744133117000251

47. Czaika M, De Haas H. Briefing: Determinants of Migration to the UK 2017 [Available from: http://migrationobservatory.ox.ac.uk/wp-content/uploads/2016/04/Briefing-Determinants-of-Migration-17-1.pdf.

48. Home Office, UK Visas and Immigration. Immigration Act: overview 2016 [updated 12 July 2016. Available from: https://www.gov.uk/government/publications/immigration-bill-2015-overarching-documents/immigration-bill-201516-overview-factsheet accessed 18 October 2019.

49. Home Office. Immigration Act 2016: Factsheet – Support for certain categories of migrants (Section 66) 2016 [updated July 2016. Available from: https://assets.publishing.service.gov.uk/government/uploads/system/uploads/attachment_data/file/537248/Immigration_Act_-_Part_5_-_Support_for_Certain_Categories_of_migrants.pdf.

50. Liberty (The National Council for Civil Liberties). Liberty's written evidence to the Public Health England consultation on the public health impact of the Memorandum of Understanding on data sharing between NHS Digital, the Home Office, and the Department of Health and Social Care 2018 [Available from: https://www.libertyhumanrights.org.uk/sites/default/files/Libertys%20written%20evidence%20to%20the%20Public%20Health%20England%20consultation%20on%20the%20public%20health%20impact%20of%20the%20MOU%20on%20data%20sharing%20between%20NHSD%20HO%20DHSC_0.pdf accessed 30 May 2019.

51. The United Kingdom Caldicott Guardian Council. [Available from: https://www.ukcgc.uk/ accessed 22 October 2018.

52. Saadi A, Ahmed S, Katz MH. Making a Case for Sanctuary Hospitals. *JAMA* 2017;318(21):2079-80. doi: 10.1001/jama.2017.15714

53. Health and Social Care Committee. Memorandum of understanding on datasharing between NHS Digital and the Home Office: Fifth Report of Session 2017–19: House of Commons, 2018:1-33.

54. Bates E. Safe surgeries: how Doctors of the World are helping migrants access healthcare. *BMJ* 2019;364

55. Charmaz K, Thornberg R, Keane E. Chapter 18: Evolving Grounded Theory and Social Justice Inquiry. In: Denzin NK, Lincoln YS, eds. The SAGE Handbook of Qualitative Research. 5th ed: SAGE Publications 2018:411-43.

56. Lincoln YS, Guba EG. Naturalistic Inquiry: SAGE Publications 1985:363-364.

For peer review only - http://bmjopen.bmj.com/site/about/guidelines.xhtml

57. Rafighi E, Poduval S, Legido-Quigley H, et al. National Health Service Principles as Experienced by Vulnerable London Migrants in "Austerity Britain": A Qualitative Study of Rights, Entitlements, and Civil-Society Advocacy. *Int J Health Policy Manag* 2016;5(10):589-97. doi: 10.15171/ijhpm.2016.50

58. Brinkmann S, Kvale S. InterViews: Learning the Craft of Qualitative Research Interviewing. 3rd ed: SAGE Publications 2015.

59. Glaser BG, Strauss AL. The Discovery of Grounded Theory: Strategies for Qualitative Research: Aldine 1967.

60. Scholl J. Escaping the Conceptual Analysis Straightjacket: Pathological Mechanisms and Canguilhem's Biological Philosophy. *Perspectives in Biology and Medicine* 2015;58(4):395–418.

61. O'Brien BC, Harris IB, Beckman TJ, et al. Standards for Reporting Qualitative Research: A Synthesis of Recommendations. *Academic Medicine* 2014;89(9):1245-51. doi: 10.1097/acm.0000000000000388

62. Doctors of the World UK. Safe Surgeries Toolkit 2018 [Available from: https://www.doctorsoftheworld.org.uk/safe-surgeries-toolkit accessed 28 May 2018.

63. Health and Social Care Committee. Memorandum of understanding on data-sharing inquiry 2018 [Available from: https://www.parliament.uk/business/committees/committees-a-z/commons-select/health-committee/inquiries/parliament-2017/mou-data-sharing-nhs-digital-home-office-inquiry-17-19/ accessed 20 November 2018.

64. McKee M. Lessons from the Windrush generation. *BMJ* 2018;361

65. General Medical Council. Confidentiality: good practice in handling patient information 2017 [updated 25 May 2018. Available from: https://www.gmc-uk.org/-/media/documents/confidentiality-good-practice-in-handling-patient-information---english-0417_pdf-70080105.pdf accessed 28 May 2018.

66. Foucault M. The Subject and Power. *Critical Inquiry* 1982;8(4):777-95. doi: 10.1086/448181

67. Oral evidence: Memorandum of understanding on data-sharing. Health and Social Care Committee. HC 677 ed: House of Commons., 2018.

For peer review only

For peer review only - http://bmjopen.bmj.com/site/about/guidelines.xhtml

TABLES

Table 1: Summary of study participants' demographics..10

FIGURE LEGENDS

Figure 1: A timeline of the Memorandum of Understanding (or MOU) between the UK Home Office/NHS Digital formalising a sharing of non-clinical patient data for immigration enforcement purposes……………………………………………...……………………………4

Figure 2: A conceptual thematic framework of key themes and sub-themes identified through data analysis used to organise findings. The framework also explores each theme's relationship with the NHS principles and at an individual (micro), organisational (meso) and societal (macro) level ……………………………………...……………...……………10

Figure 3: The patient pathway for primary care services and the perceived barriers migrant patients may face as described by participants and the literature. Perceived barriers highlighted in **bold** were described during interviews …………………………………………...12

For peer review only

Figure 1: A timeline of the Memorandum of Understanding (or MOU) between the UK Home Office/NHS Digital formalising a sharing of non-clinical patient data for immigration enforcement purposes.

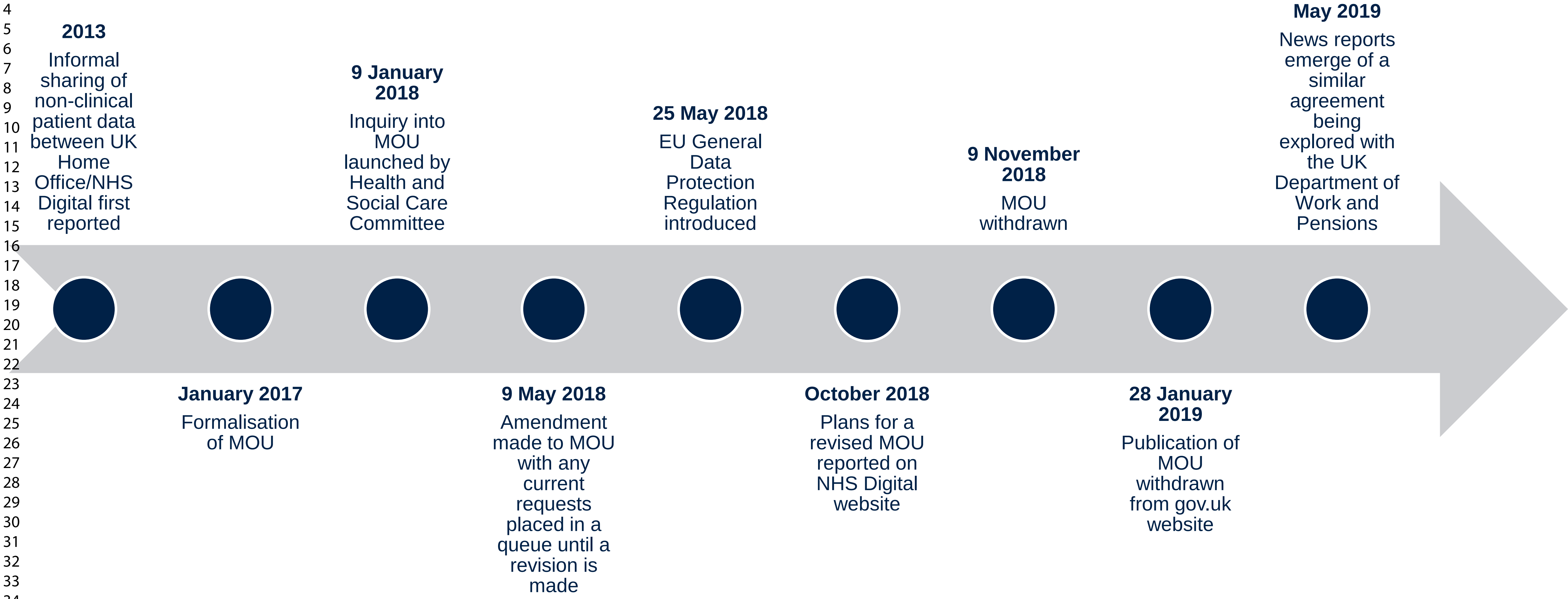

Figure 2: A conceptual thematic framework of key themes and sub-themes identified through data analysis used to organise findings. The framework also explores each themes relationship within the NHS principles and at an individual (micro), organisational (meso) and societal (macro) level.

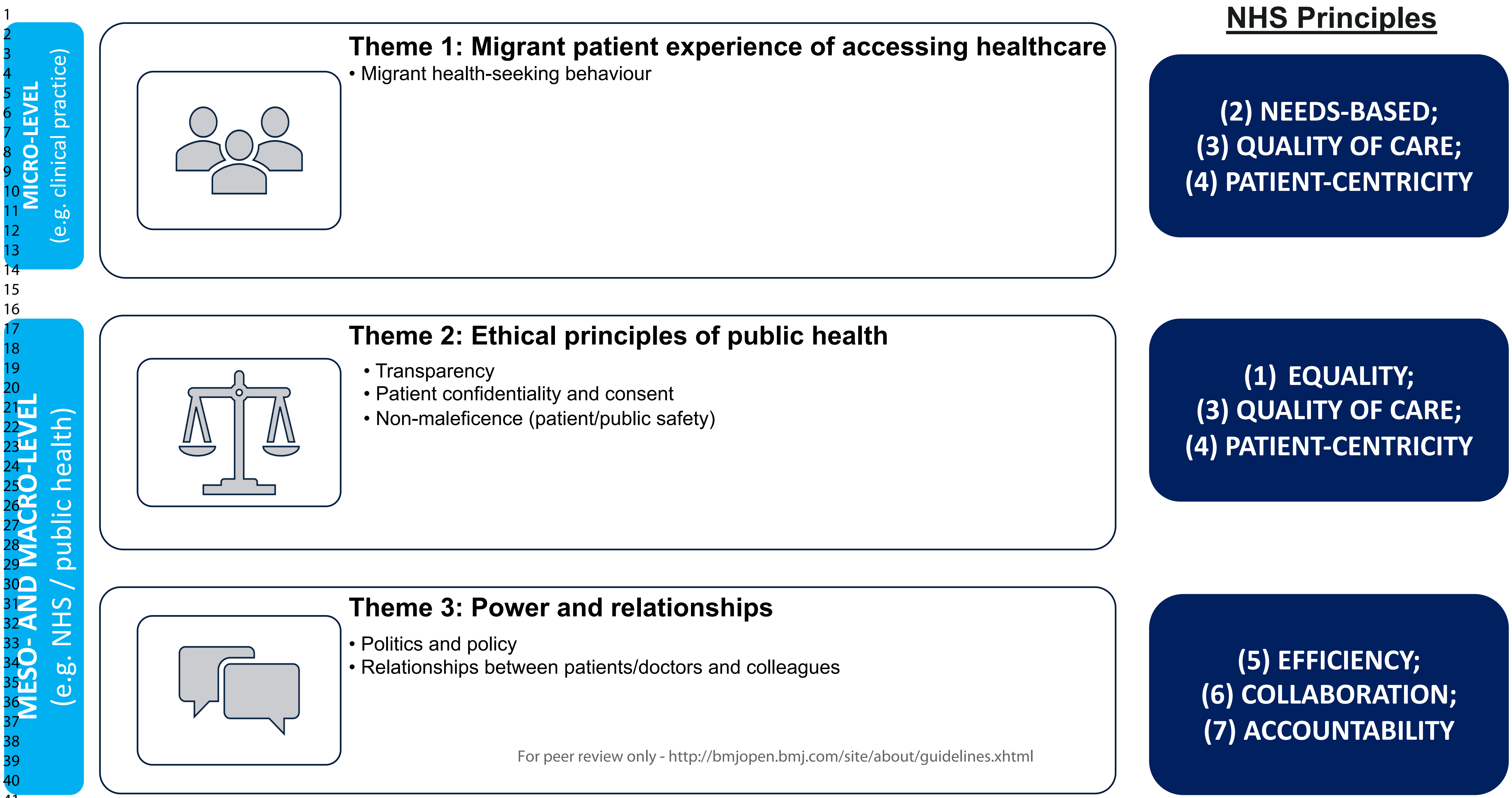

For peer review only - http://bmjopen.bmj.com/site/about/guidelines.xhtml

Figure 3: The patient pathway for primary care services and the perceived barriers migrant patients may face as described by participants and the literature. Perceived barriers highlighted in **bold** were described during interviews.

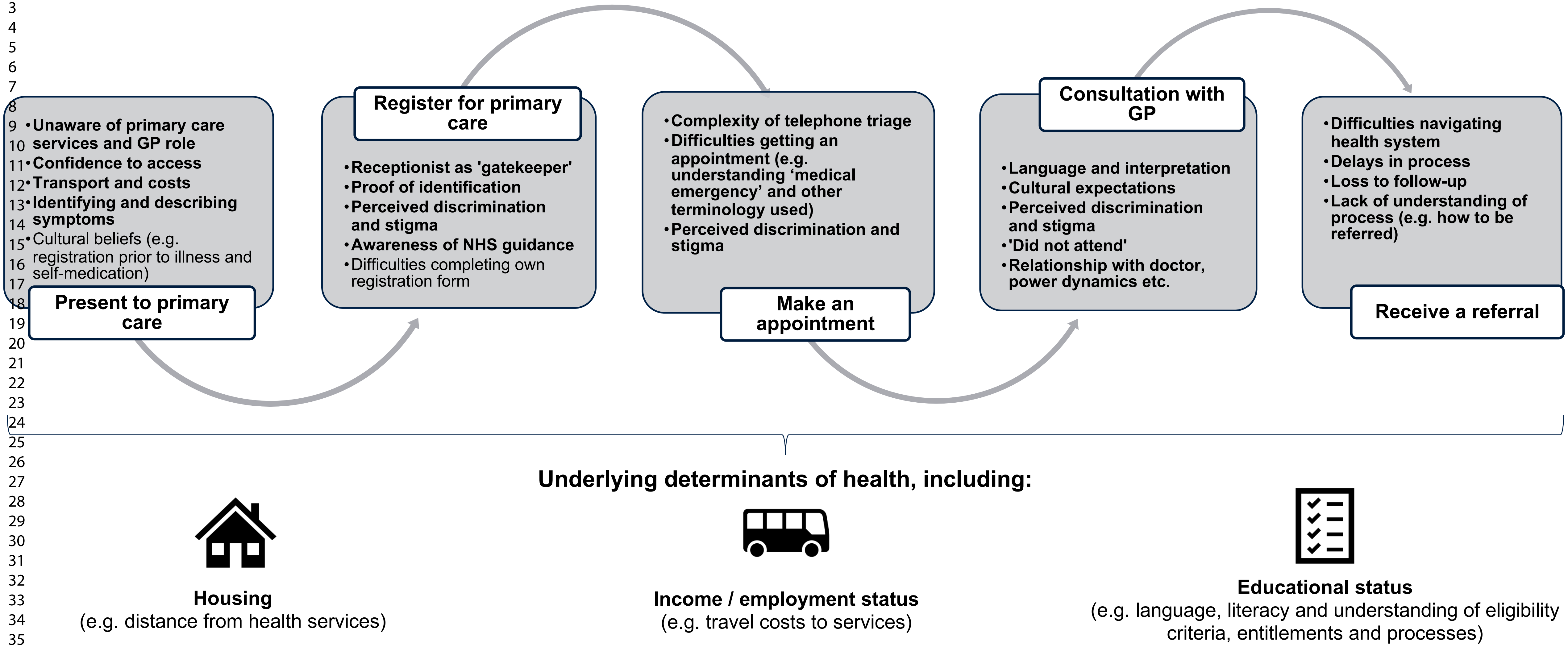

Abbreviations: GP, General Practice or Practitioner; NHS, National Health Service

Supplementary Appendix 1: Non-clinical data shared in accordance to the MoU, including if individual is deceased. Source: NHS Digital.

Non-clinical data shared

- Name (and date last known to be effective)
- Gender
- Date of birth (and date last known to be effective)
- Address
- Primary care support area code
- Primary care support area name
- Date of GP/primary care registration
- Primary care support England contact details (email and telephone number)

Data shared (if deceased)

- Name (and date last known to be effective)
- Date of birth
- Death registration district
- Death registration year/quarter

REFERENCE: NHS Digital. Written evidence from NHS Digital (MOU0004): House of Commons, 2018.

For peer review only

Supplementary Appendix 2: Interview discussion topic guide

Introductory Questions

1. Can you please tell me more about your organisation and your role?

2. How many years have you been working in this role?

3. How many years have you been practicing medicine?

Theme 1: Awareness, understanding, perceptions of patient data sharing, as described in the Home Office, Department of Health, NHS Digital MoU

1. Does your practice /organisation collect demographic data about people who come to access services? If so, please could you tell me the type of data that is collected and recorded? (e.g. name, DOB, country of birth, NHS number, current address (is this mandatory?), telephone number)

2. Outside of the NHS system, who may have access to patient demographic data?

3. Who do you think should have access to patient demographic data, outside of the NHS? Prompts: Why? Why Not?

4. What can you tell me about NHS Digital sharing data with the Home Office?

***ONLY** if they **do not know anything** about it, read out: "Since 2013, NHS Digital had been informally sharing patient administrative data, such as name, date of birth and address with the Home Office on an ad hoc basis to assist the Home Office with locating and prosecuting migrants in relation to immigration issues. That process was formalised in a Memorandum of Understanding in January 2017. This agreement was amended in May 2018. "*

a. How did you hear about this data sharing arrangement?

b. When do you first remember hearing about it?

c. What do you think about this policy? (of NHS Digital sharing demographic data with the Home Office in order for the Home Office to investigate immigration offences; previously any offenders but now only individuals convicted of, or under investigation for, serious crime?)

5. Has your awareness about this data sharing influenced how your practice / organisation records patient administrative data? If so, how?

6. Has knowledge of the data sharing changed the advice you give to patients? If so, how?

7. How was the data sharing viewed by your colleagues? Organisation? and peers?

8. Can you tell me about any interactions that you have had with patients about the data sharing?

9. Based on your experience, did knowledge that administrative data was being shared between NHS Digital and the Home Office have implications for how patients felt about accessing your services? How so?

For peer review only

Theme 2: Experience with migrant patients

10. In your work as XXX (e.g. GP), do you see patients who have been born in a country other than the UK, i.e. migrants?

11. Can you tell me more about this patient population? Prompts: Country of origin, English language ability

12. What have your experiences been like with migrant patients? (i.e. compared to UK born patients)?

13. From your experience, what can you tell me about their uptake of primary health services? Compared to British patients?

14. In your experience, is there a distinct different with migrant patients' health needs?

15. Thinking about your own experience, are you aware of any barriers that migrants might face in accessing services? Can you expand on what these might be and why?

16. From your experience, have migrant patients expressed any concerns about their data being shared with the Home Office, or concerns that accessing health services may lead to immigration authorities contacting them?

17. Thinking about this issue, has the data sharing affected your relationship with migrant patients?

Theme 3: Patient confidentiality

18. As a clinician, how you do view this data sharing in relation to patient confidentiality? Prompts: Why? How?

19. How do you think the NHS Digital data sharing may be perceived by the public? Specifically, for example public trust in NHS' management and guardianship of patient data?

Theme 4: Patient safety

IF participant cites fear of immigration or data sharing as a barrier for migrants' uptake of health services:

20. In your opinion, what are the implications of the data sharing for patient safety? For migrant patients? For vulnerable populations such as pregnant women, children, people with infectious diseases?

21. Lastly, what do you think of the data sharing in the broader terms of the general public health?

Thank you for your time. Do you have any questions for me?

****END RECORDING****

For peer review only

Supplementary Appendix 3: The 7 NHS Principles of the NHS Constitution for England. Source: Department of Health and Social Care.

NHS Principle	Summarised term
1. Provide comprehensive service, available to all	Universal access (equality)
2. Access based on clinical need, not individual's ability to pay	Needs-based
3. Aspires to the highest standards of excellence and professionalism	Quality of care
4. Patient at the heart of everything NHS does	Patient-centricity
5. Working across organisational boundaries	Collaboration
6. Provides best value for taxpayer's money	(Economic) Efficiency
7. Accountable to public, communities and patients it serves	Accountability

REFERENCE: Department of Health and Social Care. *The NHS Constitution for England*. [Updated October 2015] Available from: https://www.gov.uk/government/publications/the-nhsconstitution-for-england/the-nhs-constitution-for-england [Accessed: 17th May 2018].

For peer review only

Standards for Reporting Qualitative Research (SRQR)

Manuscript

Patient data sharing for immigration enforcement: a qualitative study of healthcare providers in England

Number and Topic	Item	Page number
**Title and abstract**		
S1 Title	Concise description of the nature and topic of the study Identifying the study as qualitative or indicating the approach (e.g., ethnography, grounded theory) or data collection methods (e.g., interview, focus group) is recommended	**1**
S2 Abstract	Summary of key elements of the study using the abstract format of the intended publication; typically includes background, purpose, methods, results, and conclusions	**2**
**Introduction**		
S3 Problem formulation	Description and significance of the problem/phenomenon studied; review of relevant theory and empirical work; problem statement	**4-6**
S4 Purpose or research question	Purpose of the study and specific objectives or questions	**6**
**Methods**		
S5 Qualitative approach and research paradigm	Qualitative approach (e.g., ethnography, grounded theory, case study, phenomenology, narrative research) and guiding theory if appropriate; identifying the research paradigm (e.g., postpositivist, constructivist/ interpretivist) is also recommended; rationale**	**7**
S6 Researcher characteristics and reflexivity	Researchers' characteristics that may influence the research, including personal attributes, qualifications/experience, relationship with participants, assumptions, and/or presuppositions; potential or actual interaction between researchers' characteristics and the research questions, approach, methods, results, and/or transferability	**7**
S7 Context	Setting/site and salient contextual factors; rationale**	**7**
S8 Sampling strategy	How and why research participants, documents, or events were selected; criteria for deciding when no further sampling was necessary (e.g., sampling saturation); rationale**	**7**
S9 Ethical issues pertaining to human subjects	Documentation of approval by an appropriate ethics review board and participant consent, or explanation for lack thereof; other confidentiality and data security issues	**8-9**
S10 Data collection methods	Types of data collected; details of data collection procedures including (as appropriate) start and stop dates of data collection and analysis, iterative process, triangulation of sources/methods, and modification of procedures in response to evolving study findings; rationale**	**7-8**
S11 Data collection instruments and technologies	Description of instruments (e.g., interview guides, questionnaires) and devices (e.g., audio recorders) used for data collection; if/how the instrument(s) changed over the course of the study	**7-8**

Developed from: O'Brien B.C., Harris, I.B., Beckman, T.J., Reed, D.A., & Cook, D.A. (2014). **Standards for reporting qualitative research: a synthesis of recommendations.** *Academic Medicine,* 89(9), 1245-1251.

For peer review only

S12 Units of study	Number and relevant characteristics of participants, documents, or events included in the study; level of participation (could be reported in results)	**10**
S13 Data processing	Methods for processing data prior to and during analysis, including transcription, data entry, data management and security, verification of data integrity, data coding, and anonymization/de-identification of excerpts	**8**
S14 Data analysis	Process by which inferences, themes, etc., were identified and developed, including the researchers involved in data analysis; usually references a specific paradigm or approach; rationale**	**7-8**
S15 Techniques to enhance trustworthiness	Techniques to enhance trustworthiness and credibility of data analysis (e.g., member checking, audit trail, triangulation); rationale**	**8**
**Results/findings**		
S16 Synthesis and interpretation	Main findings (e.g., interpretations, inferences, and themes); might include development of a theory or model, or integration with prior research or theory	**10-15**
S17 Links to empirical data	Evidence (e.g., quotes, field notes, text excerpts, photographs) to substantiate analytic findings	**10-15**
**Discussion**		
S18 Integration with prior work, implications, transferability, and contribution(s) to the field	Short summary of main findings; explanation of how findings and conclusions connect to, support, elaborate on, or challenge conclusions of earlier scholarship; discussion of scope of application/generalizability; identification of unique contribution(s) to scholarship in a discipline or field	**16-17**
S19 Limitations	Trustworthiness and limitations of findings	**16-17**
**Other**		
S20 Conflicts of interest	Potential sources of influence or perceived influence on study conduct and conclusions; how these were managed	**19**
S21 Funding	Sources of funding and other support; role of funders in data collection, interpretation, and reporting	**19**

* The authors created the SRQR by searching the literature to identify guidelines, reporting standards, and critical appraisal criteria for qualitative research; reviewing the reference lists of retrieved sources; and contacting experts to gain feedback. The SRQR aims to improve the transparency of all aspects of qualitative research by providing clear standards for reporting qualitative research.

**The rationale should briefly discuss the justification for choosing that theory, approach, method, or technique rather than other options available, the assumptions and limitations implicit in those choices, and how those choices influence study conclusions and transferability. As appropriate, the rationale for several items might be discussed together.

Developed from: O'Brien B.C., Harris, I.B., Beckman, T.J., Reed, D.A., & Cook, D.A. (2014). **Standards for reporting qualitative research: a synthesis of recommendations.** *Academic Medicine*, 89(9), 1245-1251.

For peer review only
